# Isomer-Specific Monitoring of Sialylated *N*-Glycans Reveals Association of α2,3-Linked Sialic Acid Epitope With Behcet’s Disease

**DOI:** 10.3389/fmolb.2021.778851

**Published:** 2021-11-23

**Authors:** Nari Seo, Hyunjun Lee, Myung Jin Oh, Ga Hyeon Kim, Sang Gil Lee, Joong Kyong Ahn, Hoon-Suk Cha, Kyoung Heon Kim, Jaehan Kim, Hyun Joo An

**Affiliations:** ^1^ Graduate School of Analytical Science and Technology, Chungnam National University, Daejeon, South Korea; ^2^ Asia Glycomics Reference Site, Daejeon, South Korea; ^3^ Department of Food and Nutrition, Chungnam National University, Daejeon, South Korea; ^4^ Division of Rheumatology, Department of International Medicine, Kangbuk Samsung Hospital, Sungkyunkwan University School of Medicine, Seoul, South Korea; ^5^ Division of Rheumatology, Department of Medicine, Samsung Medical Center, Sungkyunkwan University School of Medicine, Seoul, South Korea; ^6^ Department of Biotechnology, Graduate School, Korea University, Seoul, South Korea

**Keywords:** Behcet’s disease, glycan isomer, sialic acid epitope, quantitation, biomarker

## Abstract

Behcet’s disease (BD) is an immune disease characterized by chronic and relapsing systemic vasculitis of unknown etiology, which can lead to blindness and even death. Despite continuous efforts to discover biomarkers for accurate and rapid diagnosis and optimal treatment of BD, there is still no signature marker with high sensitivity and high specificity. As the link between glycosylation and the immune system has been revealed, research on the immunological function of glycans is being actively conducted. In particular, sialic acids at the terminus of glycoconjugates are directly implicated in immune responses, cell–cell/pathogen interactions, and tumor progression. Therefore, changes in sialic acid epitope in the human body are spotlighted as a new indicator to monitor the onset and progression of immune diseases. Here, we performed global profiling of *N*-glycan compositions derived from the sera of 47 healthy donors and 47 BD patients using matrix-assisted laser desorption/ionization-time of flight mass spectrometry (MALDI-TOF MS) to preferentially determine BD target glycans. Then, three sialylated biantennary *N*-glycans were further subjected to the separation of linkage isomers and quantification using porous graphitized carbon-liquid chromatography (PGC-LC)/multiple reaction monitoring (MRM)-MS. We were able to successfully identify 11 isomers with sialic acid epitopes from the three glycan compositions consisting of Hex_5_HexNAc_4_NeuAc_1_, Hex_5_HexNAc_4_Fuc_1_NeuAc_1_, and Hex_5_HexNAc_4_NeuAc_2_. Among them, three isomers almost completely distinguished BD from control with high sensitivity and specificity with an area under the curve (AUC) of 0.945, suggesting the potential as novel BD biomarkers. In particular, it was confirmed that α2,3-sialic acid at the terminus of biantennary *N*-glycan was the epitope associated with BD. In this study, we present a novel approach to elucidating the association between BD and glycosylation by tracing isomeric structures containing sialic acid epitopes. Isomer-specific glycan profiling is suitable for analysis of large clinical cohorts and may facilitate the introduction of diagnostic assays for other immune diseases.

## Introduction

Behcet’s disease (BD) is a rare immune disorder of chronic multisystemic vasculitis that causes recurrent oral and genital ulcers with skin, eye, gastrointestinal, neurological, and articular involvement (Batu and Ozen, 2012; Jennette et al., 2013; Chen and Yao, 2021). Although BD does not immediately lead to death, the gradual progression of the disease leads to serious disability with complications, so early detection and steady management of BD are emphasized. About 18 different clinical criteria have been used for the diagnosis and management of BD (Batu, 2019), and it is still difficult to determine BD because the clinical features vary greatly depending on the patients’ age, ethnicity, gender, and country (International Team for the Revision of the International Criteria for Davatchi et al., 2014; Alpsoy, 2016; Kone-Paut, 2016). To date, HLA-B*51, a split allele of human leukocyte antigen (HLA) B5, has been recognized as a potent genetic marker for the onset of BD, but whether it is a general susceptibility gene remains uncertain (Suzuki Kurokawa and Suzuki, 2004; Khabbazi et al., 2019). Therefore, the demand for pathological biomarkers from a new perspective that enables accurate diagnosis and early response to BD is increasing significantly.

**GRAPHICAL ABSTRACT:**
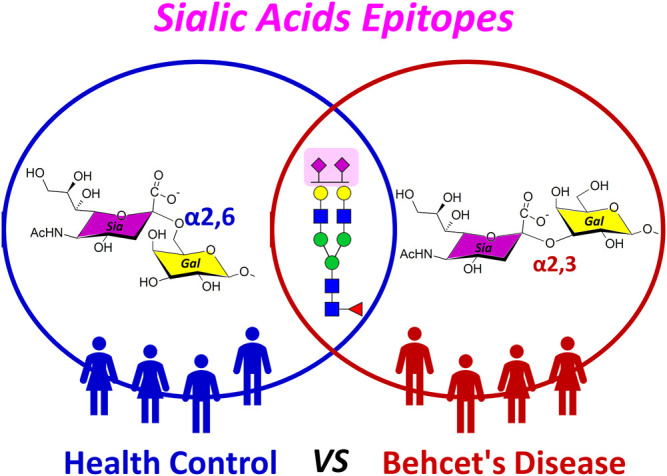


In immune systems, glycosylation has been shown to be closely related to host–pathogen interactions, immunological recognition and activation, and induction of differentiation between self and non-self (van Kooyk and Rabinovich, 2008; Reily et al., 2019). In particular, sialic acid motifs attached to the ends of the glycans with α2,3-, α2,6- or α2,8-linkages form the outermost identity of the cell (Hanamatsu et al., 2018). Thus, changes in sialic acid such as position, linkage, and quantity may be associated with pathophysiological conditions (Schnaar et al., 2014; Zhang et al., 2018; Klaus et al., 2021). Indeed, influenza viruses have different affinities to α2,3- or α2,6-linked sialic acids of host glycans for human infection (Nicholls et al., 2007; Yang et al., 2018). The ratio of α2,6- to α2,3-linked sialic acid of glycans in chondrocyte affects the incidence of inflammatory joint disease (Toegel et al., 2010). Changes in expression of α2,3- and α2,6-linked isomers are now widely recognized as key signs of tumor development such as colorectal (Sethi et al., 2015), breast (Alley and Novotny, 2010), and prostate cancers (Gilgunn et al., 2013; Dobie and Skropeta, 2021). Similarly, α-2,6-linked sialylated glycans in the Fc region of antibody for inflammation treatment was safer and more effective than those with α2,3-linkage (Zhang et al., 2019). Ultimately, understanding the role of sialic acid epitopes in biological processes could be an important factor in solving immunological problems.

Despite the importance of sialic acid epitopes in several health issues, accurate identification and quantification of sialylated glycans at the isomeric level remain a challenge due to the extremely heterogeneous sialyation and labile sialic acid residues (Cheng et al., 2021). Today, selection/multiple response monitoring (SRM/MRM) coupled with liquid chromatography (LC) is emerging as an ideal technique for glycan quantitation (Zhou et al., 2015; Seo et al., 2021). MRM is primarily performed on triple quadrupole (QqQ) instruments, which profile a pair of precursor/fragment ions called transitions (Ruhaak and Lebrilla, 2015; Veillon et al., 2017). High selectivity and high sensitivity are achieved due to two selection stages to filter out co-eluted background ions. It can also allow for linear response over a wide dynamic range for much lower amounts of compounds in complex mixtures (Hong et al., 2013; Hong et al., 2014). In addition, porous graphitized carbon (PGC) columns are widely used for high-resolution separation of complex and heterogeneous native and reduced glycans (Hua et al., 2011; Hua et al., 2013; Huang et al., 2017; Abrahams et al., 2018). In fact, several studies applying PGC-LC/MRM-mass spectrometry (MS) have been reported in various fields of glycomics (Wang et al., 2017; Xu et al., 2017; Flowers et al., 2019). Namely, PGC-LC/MRM-MS is a more stable and reliable method with various benefits for investigating the isomers of sialylated glycan associated with immune diseases.

Here, we selectively monitored glycan isomers with different sialic acid epitopes from the sera of 47 healthy individuals with no clinical history and 47 patients diagnosed with BD using PGC-LC/MRM-MS. A total of 11 isomers corresponding to three targeted sialylated biantennary glycans, Hex_5_HexNAc_4_NeuAc_1_, Hex_5_HexNAc_4_Fuc_1_NeuAc_1_, and Hex_5_HexNAc_4_NeuAc_2_, were identified in all clinical samples. We confirmed that three isomers are associated with BD-specific α2,3-sialic acid epitope, which could ultimately be potential biomarkers for BD diagnosis [area under the curve (AUC) > 0.945]. Interestingly, in the control group, more α2,3-sialic acid binding isomers were observed in females than in males, suggesting that the specific epitope is gender related (*p* < 0.001, Student’s *t*-test). In this study, we presented that the sialic acid epitope in blood could be identified simply and quickly through isomer-specific monitoring of sialylated glycans. In addition, this study can be utilized for various immune-related studies, including disease diagnosis, disease activity evaluation, and disease pathogenesis in which sialic acid epitope is involved.

## Materials and Methods

### Reagents

Peptide *N-*glycosidase F (PNGase F) and α2,3 neuraminidase were purchased from New England Biolabs (MA, United States). PGC cartridges were obtained from Agilent Technologies (Santa Clara, CA, United States). All solvents such as water, acetonitrile (Sigma-Aldrich, St. Louis, MO, United States), and formic acid (Honeywell, Charlotte, NC, United States) were LC/MS grade. Other materials and reagents were of analytical grade or higher unless otherwise noted below.

### Serum Samples

A total of 94 sera were obtained from the rheumatology clinic at the Samsung Medical Center and Kangbuk Samsung Hospital in Seoul, Korea. To minimize potential confounding effects, 47 patients had Behcet’s disease, and age- and sex-balanced 47 healthy volunteers served as the healthy control group. Patients met all the recruitment requirements in accordance with the criteria of the 1990 International Study Group (ISG) for Behcet’s disease. The experimental protocols used in this study were approved by the Samsung Medical Center (#2014-01-082) and Kangbuk Samsung Hospital Institutional Review Board (#KBC14082), and written informed consent was obtained from each patient enrolled in this study. The donor information is briefly described in Supplementary Table S1.

### Enzymatic Release of *N-*Glycans From Serum

To release *N-*glycans from human serum, 50 μl of serum samples were initially mixed with 50 μl of digestion buffer (pH 7.5, 100 mM ammonium bicarbonate and 5 mM dithiothreitol) and then thermally denatured between a 100°C and 20°C water bath for six cycles of 20 s each. After it was cooled, 2 μl of PNGase F (1,000 units) was added, and the mixture was incubated in a water bath at 37°C for 16 h. Deglycosylated proteins were precipitated out with chilled ethanol, leaving a glycan-rich supernatant that was dried in vacuum (Kronewitter et al., 2009).

### Fractionation of Sialylated *N*-Glycans

Released *N-*glycans were purified and fractionated using porous graphitized carbon-solid phase extraction (PGC-SPE) according to molecule size and polarity. Briefly, PGC cartridge (250 mg bed weight and 6 ml cartridge volume) was conditioned with water followed by 80% acetonitrile (ACN) with 0.1% trifluoroacetic acid (TFA) in water (*v*/*v*). Glycan solution reconstituted with 1 ml of water was loaded to PGC cartridge and subsequently washed with water at a flow rate of approximately 1 ml/min to remove excess salts and buffer. The *N-*glycans were fractionated stepwise in the following order: 1) 10% ACN in water (*v*/*v*), 2) 20% ACN in water (*v*/*v*), and 3) 40% ACN with 0.05% TFA in water (*v*/*v*). Finally, each SPE fraction was absolutely dried in vacuum.

### MALDI-TOF/TOF MS Analysis

Rapid mass profiling of serum *N*-glycans was performed using a previously optimized procedure (Oh et al., 2013; Ji et al., 2015). Briefly, each SPE fraction was spotted onto a stainless steel matrix-assisted laser desorption/ionization (MALDI) plate with an equal volume of 2,5-dihydroxybenzoic acid (DHB) solution (5 mg/100 μl in 50% ACN/H_2_O) and then dried under vacuum prior to loading into an ultrafleXtreme MALDI-time-of-flight/time-of-flight (TOF/TOF) system (Bruker, Billerica, MA, United States). Glycans eluted by 1) 10% ACN in water (*v*/*v*) and 2) 20% ACN in water (*v*/*v*) were analyzed in positive ion reflectron mode, while glycans eluted by 3) 40% ACN with 0.05% TFA in water (*v/v*) were analyzed in negative ion reflectron mode. Mass spectra were recorded over a range of *m*/*z* 500–5,000.

### PGC-LC/MRM-MS Analysis

Dried sialylated *N*-glycans were reconstituted by 50 μl of water and then analyzed using Agilent 1260 LC system coupled to an Agilent 6460 triple quadrupole MS (Agilent Technologies, Santa Clara, CA). A Thermo Hypercarb™ column (3 mm i.d., 100 mm length, and 3 µm particle size) was used for LC separation. Upon injection of 2 μl of sample, sialylated *N*-glycans were chromatographically separated for 45 min using binary gradient consisting of solvent A of 3% ACN with 0.1% FA (*v*/*v*) and solvent B of 90% ACN with 0.1% FA (*v*/*v*) at a flow rate of 0.3 ml/min. The LC gradient used was as follows: 0–0.25 min, 7%, 0.3 ml/min B; 0.25–10 min, 7%–15%, 0.3 ml/min B; 10–25 min, 15%–40%, 0.3 ml/min B; 25–30 min, 40%–100%, 0.3 ml/min B; 30–35 min, 100%, 0.3 ml/min B; 35–35.1 min, 100%–7%, 0.3 ml/min B; and 35.1–45 min, 7%, 0.3 ml/min B. MS was operated in the positive mode. The first and third quadrupoles were operated at unit resolution. The optimal parameters used were as follows: dry gas temperature 250°C, dry gas flow 10 l/min, nebulizer pressure 30 psi, sheath gas temperature 300°C, sheath gas flow 12 l/min, and capillary voltage 4,000 V. MRM transitions monitored for the study are listed in Supplementary Table S2.

### Data Processing and Statistical Analysis

Raw MALDI-TOF/TOF MS data were processed with Bruker FlexAnalysis (version 3.3). All LC/MS data was controlled and synchronized by the Agilent MassHunter Qualitative Analysis software (B.07.00) and Agilent MassHunter Quantitative Analysis software (B.06.00). The peak areas were filtered and integrated with a signal-to-noise ratio of 10. The normalized absolute peak intensity (NAPI) was calculated as follows:

$$NAPI\;=\;\frac{\left(absolute\;peak\;\mathrm{int}ensity\right)}{\left(\sum absolute\;peak\;\mathrm{int}ensity\right)}\times 100$$

Data with replicates were reported as average ± standard deviation (SD). To investigate associations between sialylated glycan isomers and Behcet’s disease, we applied multiple statistical models. The Student’s *t*-test and partial least-squares discriminant analysis (PLS-DA) were generated by R package MetaboAnalystR 3.0 to compare the quantitative level of sialylated glycan isomers between Behcet’s disease patient and healthy control. The area under a receiver operating characteristic curve (AUC-ROC) was calculated to determine the classification efficiency of Behcet’s disease biomarkers. The scatter plots were provided in GraphPad Prism (version 5.0). Pearson correlation coefficient-based scatter plot matrices were calculated using R package psych (version 3.6.1).

## Results and Discussion

**FIGURE 1:**
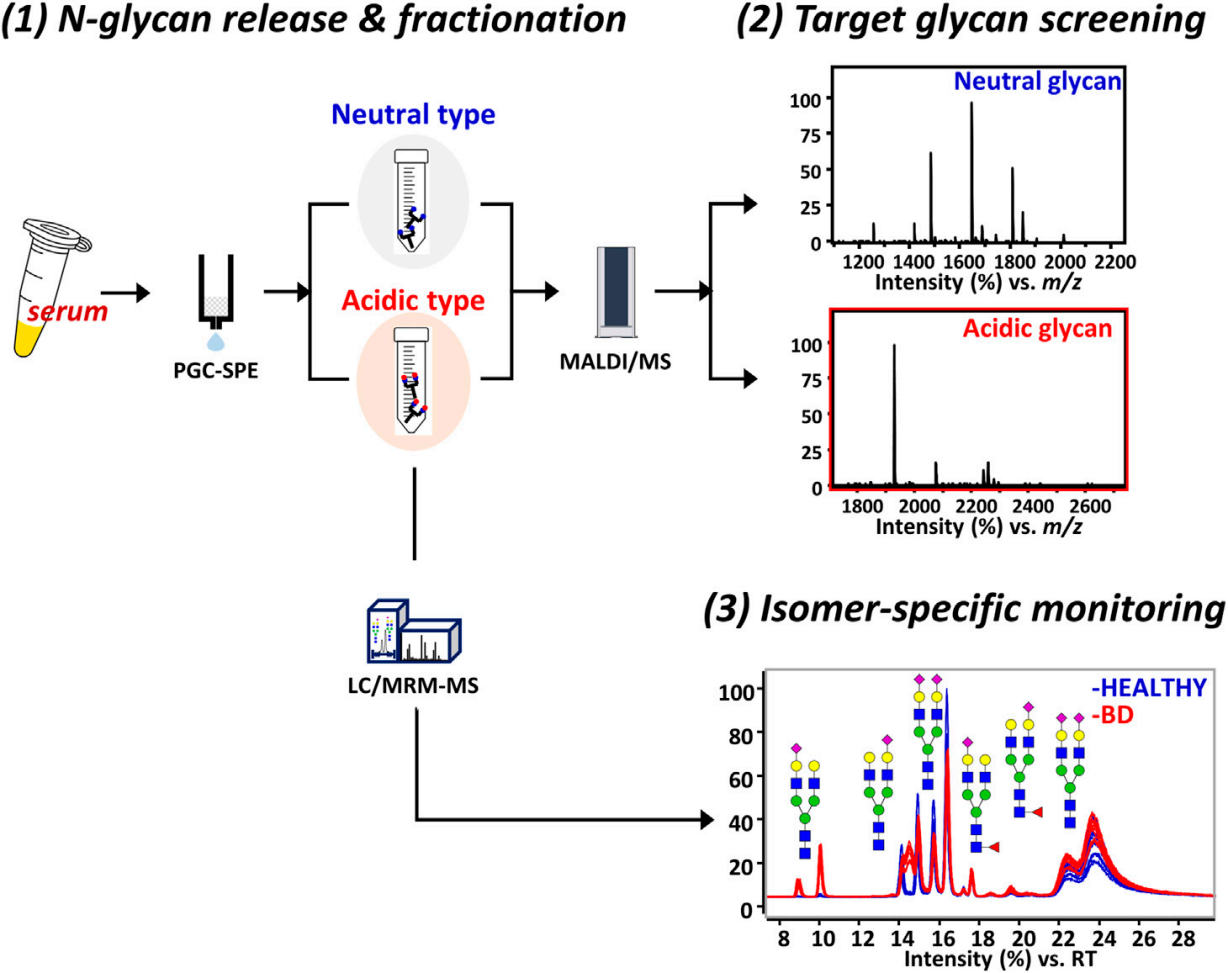
Schematic illustration of the experimental workflow for isomer-specific monitoring. The platform consists of three steps including 1) *N*-glycan fractionation, 2) mass profiling of glycans, and 3) LC/MRM-MS analysis of glycans with BD-specific epitopes. For the glycan cartoons, green circles denote mannose, yellow circles denote galactose, blue squares denote *N*-acetylglucosamine, red triangles denote fucose, and purple diamonds denote *N*-acetylneuraminic acid. LC, liquid chromatography; MRM, multiple reaction monitoring; MS, mass spectrometry; BD, Behcet’s disease.

A streamlined experimental workflow showing a step-by-step strategy for discovering BD-specific glycan epitopes is shown in Figure 1. Initially, *N*-glycans were enzymatically released in the sera of 47 healthy individuals with no clinical history and 47 patients diagnosed with BD and then separated into acidic and neutral glycans by SPE-PGC. To determine BD-specific glycans, glycan mass profiling was performed using MALDI-TOF/TOF MS. A total of 32 glycan compositions of neutral and acidic types were obtained as listed in Supplementary Table S3. The number of native sialylated glycans analyzed by MALDI-MS was lower than that obtained from derivatized glycan analysis (Jansen et al., 2016; de Vroome et al., 2018; Dedova et al., 2019), but bi- and triantennary sialylatyed glycans exhibiting the characteristics of serum glycosylation were readily identified as key molecules with high reproducibility. Interestingly, only complex glycans containing sialic acid showed statistically significant differences in the BD patient group compared to the control group. In particular, there are three sialylated glycans markedly altered in BD, all of which are biantennary glycan structure with *N*-acetylneuraminic acid (NeuAc), accounting for more than 95% of the total acidic glycans (*p* < 0.001, Student’s *t*-test). These correspond to Hex_5_HexNAc_4_NeuAc_1_, Hex_5_HexNAc_4_Fuc_1_NeuAc_1_, and Hex_5_HexNAc_4_NeuAc_2_. To gain detailed information on BD-specific glycan epitopes, three important sialylated glycans were further isolated and quantified using PGC-LC/MRM-MS.

### Isomer-Specific Separation of Glycan With Different Sialic Acid Epitopes

**FIGURE 2:**
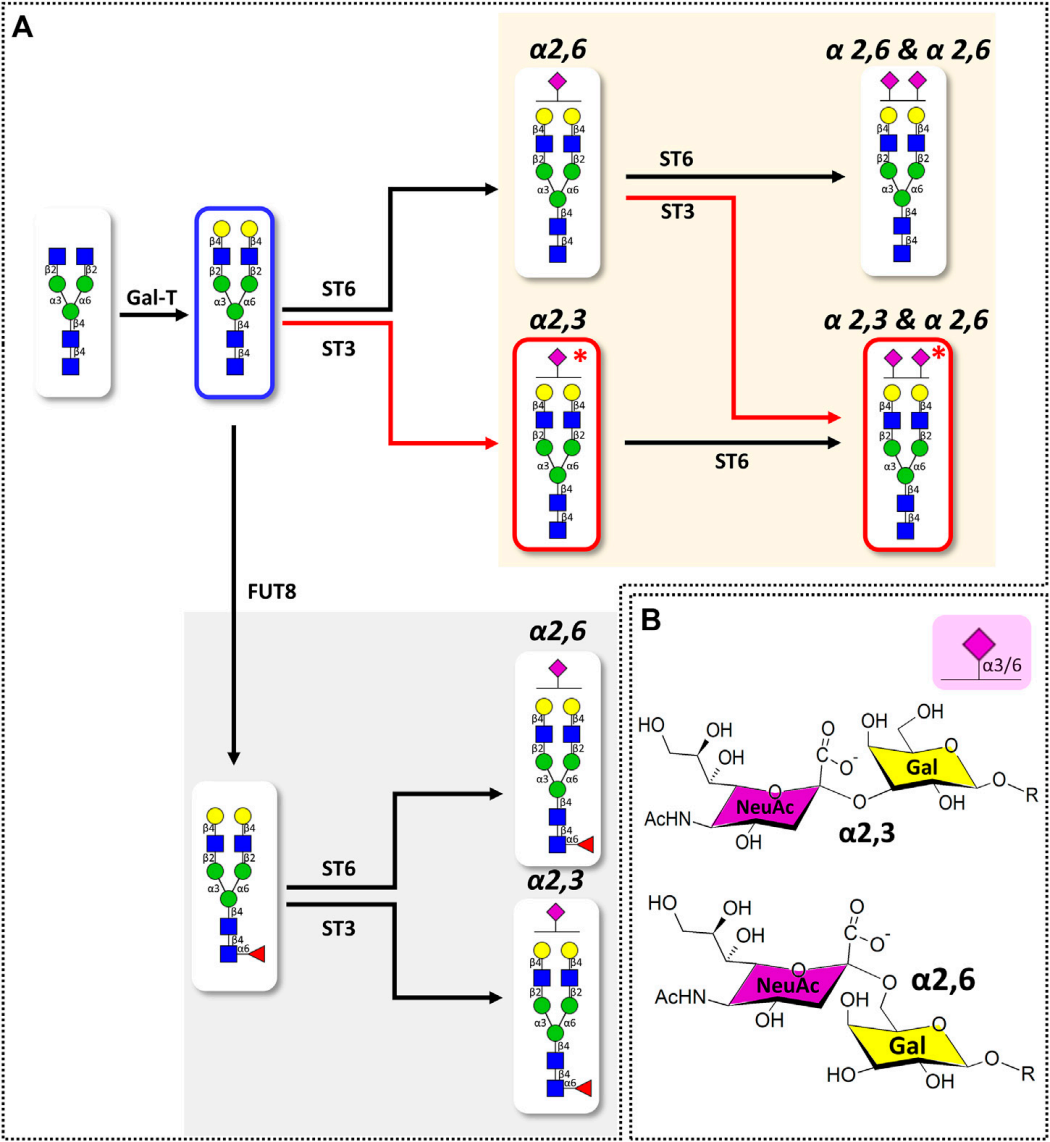
**(A)** Simplified biosynthetic pathway of target sialylated biantennary *N*-glycans and **(B)** the representative structures of terminal sialic acid epitopes in humans. Pathway and glycans associated with BD-specific sialic acid epitope are highlighted with red lines. Glycosyltransferases involved in the glycan synthesis are indicated at the top of the arrow (GalT: β1,4 galactosyltransferase; FUT8: α1,6 fucosyltransferase; ST6: α2,6 sialyltransferase; ST3: α2,3 sialyltransferase). Glycan structures were represented using SNFG nomenclature (Varki et al., 2015). Glycan schematic symbols: green circle, mannose; yellow circle, galactose; blue square, *N*-acetyl glucosamine; red triangle, fucose; purple diamonds, *N*-acetylneuraminic acid.

Glycans can have numerous isomers depending on position, linkage, branching, and anomeric structure by various feedback mechanisms of glycosidases and glycosyltransferases (Baum and Cobb, 2017), even if they are identical in composition. In particular, since three target glycans are expanded based on Hex_5_HexNAc_4_ structure in biosynthetic pathway of serum glycans (Figure 2), sialic acid capped at the non-reducing end of the common core structure can be expressed as different isomers. Therefore, an in-depth characterization of BD signature glycans *via* location, linkage, and quantitative level of sialic acid is necessary to understand the underlying mechanisms of BD disease and to discover biomarkers for diagnosis.

**FIGURE 3:**
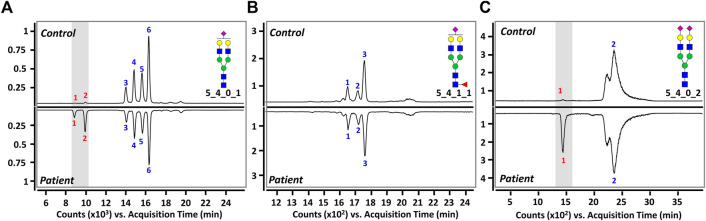
Representative PGC-LC/MRM chromatograms of **(A)** Hex_5_HexNAc_4_NeuAc_1_, **(B)** Hex_5_HexNAc_4_Fuc_1_NeuAc_1_, and **(C)** Hex_5_HexNAc_4_NeuAc_2_ in human serum. Top and bottom panels display healthy controls and BD patients, respectively. A gray bar indicates isomers with markedly different between healthy controls and patients. For the glycan cartoons, green circles denote mannose, yellow circles denote galactose, blue squares denote *N*-acetylglucosamine, red triangles denote fucose, and purple diamonds denote *N*-acetylneuraminic acid.

Three sialylated glycans were further separated and analyzed using a PGC column, which is excellent for isomer-specific separation of native glycans (Hua et al., 2011; Seo et al., 2019). Figure 3 shows the representative PGC-LC/MRM chromatograms of Hex_5_HexNAc_4_NeuAc_1_, Hex_5_HexNAc_4_Fuc_1_NeuAc_1_, and Hex_5_HexNAc_4_NeuAc_2_, respectively. We were able to achieve complete chromatographic separation isomers with identical composition (Rs > 1.5). Since the sialic acid in human glycan is primarily attached to galactose residues *via* α2,3 or α2,6 glycosidic bonds (Tsuji, 1996; Lane et al., 2019), the three glycans could theoretically have a total of 24 isomers (eight each) by sialic acid bond and α- and β-anomeric configurations. However, a total of 11 isomers corresponding to six isomers of Hex_5_HexNAc_4_NeuAc_1_, three isomers of Hex_5_HexNAc_4_Fuc_1_NeuAc_1_, and two isomers of Hex_5_HexNAc_4_NeuAc_2_ were found in both the control and BD groups. The number in each chromatogram indicates the order of isomers of each composition eluted from LC, and each isomer is denoted by “glycan composition-elution order” for convenience and clarity. The last split chromatogram peak of Hex_5_HexNAc_4_NeuAc_2_ in Figure 3C was considered as one isomer for subsequent quantitative analysis as no baseline separation was achieved. For intuitive comparison, the chromatograms of the control group and the BD patients were expressed as mirror images. In particular, isomers of Hex_5_HexNAc_4_Fuc_1_NeuAc_1_ showed high similarity in the control group and BD patients (Figure 3B), while isomers showing a significant difference were identified in the other two sialyated glycans (marked by a gray bar in (Figure 3A,C). In conclusion, the isolation and identification of isomers corresponding to three target glycans suggest that sialylated glycans containing various sialic acid epitopes are associated with BD.

### Quantitative Comparison of Sialylated Glycan Isomers Between Healthy Controls and BD Patients

For quantitative comparison between healthy controls and BD patients, an MRM analysis was performed for each isomer after chromatographic separation of sialylated glycans. Details of the MRM transitions are provided in Supplementary Table S2. By monitoring the most abundant product ions such as *m*/*z* 366.1 (GlcNAc-Gal) and *m*/*z* 274.1 (dehydrated NeuAc) generated by collision-induced dissociation (CID)-MS/MS of sialylated glycan isomers, reproducible MRM results were obtained with high sensitivity. The absolute abundances of a total of 11 isomers derived from all serum samples are listed in Supplementary Table S3. As summarized in Table 1, we were able to determine three isomers that exhibited significant quantitative changes in BD compared to controls (*p* < 10^−20^, Student’s *t*-test). Interestingly, the abundance ratio of three isomers including Hex_5_HexNAc_4_NeuAc_1_-1, Hex_5_HexNAc_4_NeuAc_1_-2, and Hex_5_HexNAc_4_NeuAc_2_-1 was significantly increased more than five times in BD compared to the control group (see *P*/*C* ratio in Table 1) (Lee et al., 2020). Additionally, in order to examine the possibility of clinical application, the AUC-ROC performance was measured (Supplementary Figure S1). Three isomers exhibited high AUC (AUC > 0.945), suggesting their potential as biomarkers with high sensitivity and high specificity. These quantitative results clearly indicate that the expression level of sialylated glycans with specific sialic acid epitopes is associated with BD.

**TABLE 1:** The absolute peak abundance and statistical analysis results of identified sialylated glycan isomers from the sera of healthy control (*n* = 47) and BD patient (*n* = 47); average values from technical replicates of each sample were presented.

Glycan isomer		Healthy control			BD patient			*p*-value	AUC^c^	*P*/*C* ratio^d^	NeuAc linkage
		Average	±	SD	Average	±	SD				
5_4_0_1^a^	1^b^	199.1	±	198.6	1,107.2	±	691.4	1.20E-25	0.946	5.6	α2,3
	2	542.1	±	499.9	2,977.1	±	1,843.5	5.27E-26	0.946	5.5	α2,3
	3	2,514.5	±	720.5	2,211.8	±	523.2	1.17E-03	0.636	0.9	α2,6
	4	5,146.5	±	1,206.9	4,724.3	±	1,197.6	1.70E-02	0.615	0.9	α2,6
	5	4,899.4	±	1,382.5	4,353.2	±	1,052.9	2.65E-03	0.631	0.9	α2,6
	6	10,153.3	±	2,273.4	9,496.0	±	2,256.3	4.81E-02	0.596	0.9	α2,6
5_4_1_1	1	811.9	±	234.5	863.6	±	260.1	1.54E-01	0.540	1.1	α2,6
	2	531.6	±	166.9	541.9	±	170.9	6.76E-01	0.516	1.0	α2,6
	3	1,967.1	±	602.3	2,150.2	±	645.4	4.58E-02	0.573	1.1	α2,6
5_4_0_2	1	746.1	±	769.1	4,723.0	±	2,661.5	1.22E-30	0.963	6.3	α2,3/α2,6 or α2,6/α2,3
	2	32,503.5	±	8,354.2	35,975.2	±	8,847.7	6.25E-03	0.634	1.1	α2,6/α2,6

^a^Four-digit glycan annotation (sugar code: #Hex_#HexNAc_#Fuc_#NeuAc).

b The eluting order of isomers of each composition on PGC-LC.

c The area under the receiver operating characteristic curve (AUC-ROC).

d The ratio between the average abundance of patient to the healthy control.

$\frac{\text{P}}{\text{C}}ratio=\frac{\left(average\;abundance\;of\;patient\right)}{\left(average\;abundance\;of\;control\right)}$

A multivariate statistical analysis was performed by PLS-DA to further confirm that healthy control and BD could be distinguished according to the expression level of isomers. Although some samples slightly overlapped in the 95% confidence interval of PLS-DA score plot (Supplementary Figure S2A), the BD and control groups were clustered and segregated, respectively (component 1 = 81.8% and component 2 = 14.1%). Significant isomers for BD screening were ranked by variable significance of projection score (VIP) derived from PLS-DA as shown in Supplementary Figure S2B. We observed that three isomers with high AUC values, Hex_5_HexNAc_4_NeuAc_1_-1, Hex_5_HexNAc_4_NeuAc_1_-2, and Hex_5_HexNAc_4_NeuAc_2_-1, had equally high VIP score (>1). The overall quantitative comparison of each isomer to the target sialylated glycans suggested that the expression of specific sialic acid epitopes was significant in BD and a key point distinguishing control and BD.

### Characterization of Sialic Acid Epitopes

**FIGURE 4:**
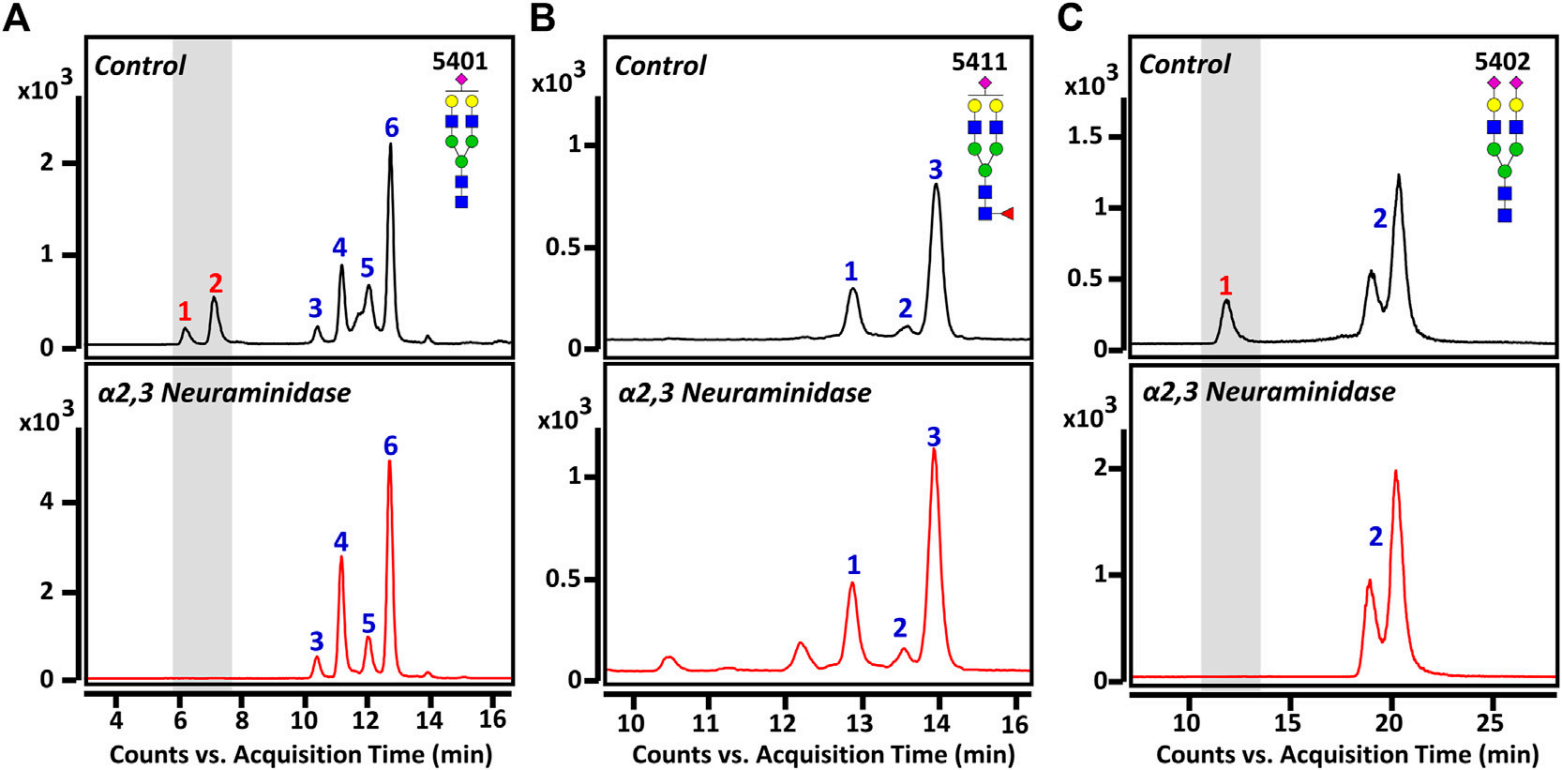
Monitoring of sialylated glycan isomers before and after exo-glycosidase digestion: **(A)** Hex_5_HexNAc_4_NeuAc_1_, **(B)** Hex_5_HexNAc_4_Fuc_1_NeuAc_1_, and **(C)** Hex_5_HexNAc_4_NeuAc_2_. The top panels indicate before treatment and bottom panels show after treatment with α2,3 neuraminidase for 3 h at 37°C, respectively. Chromatographic peaks reduced by enzymatic treatment are highlighted in gray windows. For the glycan cartoons, green circles denote mannose, yellow circles denote galactose, blue squares denote *N*-acetylglucosamine, red triangles denote fucose, and purple diamonds denote *N*-acetylneuraminic acid.

**FIGURE 5:**
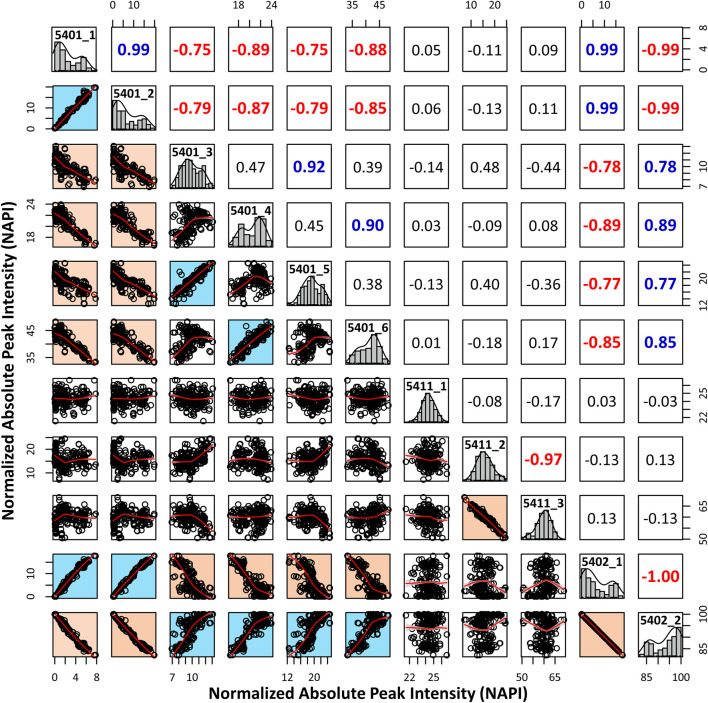
Pearson correlation analysis to investigate associations between isomers with different sialic acid epitopes. The normalized absolute peak intensities (NAPIs) of 11 isomers in all serum samples were used for calculations. The scatterplot matrix indicates the association between each isomer, and the Pearson correlation coefficient (*r*) is above the diagonal. Information for each isomer is displayed in a diagonal histogram box (isomer code: glycan composition-elution order).

We performed an in-depth characterization of sialic acid epitopes using a commercially available α2,3 neuraminidase, among several analytical strategies capable of discriminating terminal sialic acids (Bwanali et al., 2020). PGC-LC/MRM-MS profiling of sialylated glycans before and after exo-glycosidase digestion is shown in Figure 4. After the enzyme reaction, the peaks of three isomers corresponding to Hex_5_HexNAc_4_NeuAc_1_-1, Hex_5_HexNAc_4_NeuAc_1_-2, and Hex_5_HexNAc_4_NeuAc_2_-1 out of a total of 11 isomers completely diminished, confirming that the terminal sialic acid linkages were α2,3. Conversely, the isomers with no change in the peak after enzymatic treatment were assumed to have the most abundant α2,6 sialic acid linkage in human serum referring to relevant studies (Song et al., 2015; Jansen et al., 2016; de Vroome et al., 2018; Dedova et al., 2019; Reiding et al., 2019). Indeed, it was possible to confirm that the abundant isomers such as Hex_5_HexNAc_4_NeuAc_1_-4, 6 and Hex_5_HexNAc_4_NeuAc_2_-2 had α2,6 sialic acid epitopes by comparing the retention time on PGC-LC with the synthetic standards (Asparia Glycomics, San Sebastián, Spain) whose structures were fully identified (Supplementary Figure S3). The sialic acid linkage information for each isomer is listed in Table 1. A Pearson correlation analysis was performed to investigate the correlation between isomers having specific sialic acid epitopes. As shown in Figure 5, isomers having the same sialic acid epitope had a high positive correlation with each other, whereas isomers with different sialic acid epitopes showed a high negative correlation. Specifically, isomers with α2,3 sialic acid epitope showed a positive correlation with each other (*r* > 0.99), whereas with isomers with α2,6 sialic acid epitope showed a negative correlation (−0.75 < *r* < −1). Despite the isomers having the same sialic acid epitope, there was no correlation in fucose-containing isomers such as Hex_5_HexNAc_4_Fuc_1_NeuAc_1_-1, Hex_5_HexNAc_4_Fuc_1_NeuAc_1_-2, and Hex_5_HexNAc_4_Fuc_1_NeuAc_1_-3. These results may suggest that the expression of isomers with specific sialic acid epitopes sensitively responding to human physiological conditions is organically linked or competed in the glyco-synthesis process as illustrated in Figure 2.

Interestingly, we were able to further observe differences in α2,3-sialylated glycan expression between men and women in the given healthy control group set (Supplementary Figure S4). The abundance of α2,3-sialylated glycans (Hex_5_HexNAc_4_NeuAc_1_-1, Hex_5_HexNAc_4_NeuAc_1_-2, and Hex_5_HexNAc_4_NeuAc_2_-1) in BD patients was 5.5–6.3-fold higher than the healthy control group, but no difference was observed between female and male patients. However, a clear segregation has been observed between biological genders in the healthy control group showing 4.9–6.3-fold higher peak abundance of the female over the male group (top panels in Supplementary Figure S4). It must be noted that healthy females exhibited higher peak abundance than males; however, female BD patients expressed two times higher peak abundance than those in the healthy control group (bottom panels in Supplementary Figure S4). Although the molecular underpinning of sialylation, its regulatory mechanism, and target protein were not understood, it could still provide a clue of glycosylation–immune interactions as well as the genetic signature of glycosylation.

## Conclusion

As the close link between glycosylation and the immune system has been elucidated, multifaceted studies have been undertaken to explore the role of glycans in the immune system. Many efforts are being made to discover new biomarkers using glycans associated with various immune diseases, such as rheumatoid arthritis, systemic lupus erythematosus, and inflammatory bowel disease, or to elucidate the underlying mechanism of the disease. In particular, sialic acid moiety, which is located at the outermost part of the glycoconjugate and is easily exposed, is known as a key sensor for receptors of immune-related molecules involved in leukocyte trafficking, pathogen recognition, immune cell activation, and immunosuppression. Therefore, the demand for in-depth characterization of sialylated glycans in biological samples such as human blood or tissues is rapidly increasing, as the sialic acid epitope of glycans can ultimately reflect the physiological state of the body.

BD is a typical mixed-pattern disease characterized by a mixture of autoimmunity and autoinflammation, which is difficult to diagnose and treat due to various clinical symptoms and causes. Although BD is an immune-related disease, studies of its association with glycans have never been conducted. In this study, we performed isomer-specific monitoring of sialylated glycans to understand the association between BD and glycans. The specific monitoring of sialylated glycan isomers based on PGC-LC/MRM-MS enabled fast and precise quantification of glycan epitopes associated with BD without a complete elucidation of glycan structures. Indeed, for the first time, we identified sialic acid epitopes with specific expression in the serum of BD patients. In addition, potent BD glycan biomarkers (AUC > 0.945) with high sensitivity and high specificity have been found, all of which have the α2,3 sialic acid linkage of the BD-specific isomer in common. Above all, isomers with BD-specific sialic acid epitopes are in the same process line in the biosynthesis of serum glycans, suggesting a high association between BD and sialic acid epitope expression.

In summary, our analytical platform and findings will undoubtedly provide insight for identifying glycan epitopes associated with BD as well as other immune diseases and understanding the nature of diseases.

## Data Availability

The original contributions presented in the study are included in the article/Supplementary Material; further inquiries can be directed to the corresponding authors.

## Abbreviations

AUC-ROC, the area under of the receiver operating characteristic curve; BD, Behcet’s (beh-CHETS) disease; CID, collision-induced dissociation; Fuc, fucose; Hex, hexose; HLA, human leukocyte antigen; LC, liquid chromatography; m/z, mass to charge; MS, mass spectrometry; SRM/MRM, selection/multiple reaction monitoring; HexNAc, N-acetylhexosamine; NAPI, normalized absolute peak intensity; Neu5Ac, N-acetylneuraminic acid; PGC, porous graphitized carbon; PLS-DA, partial least-squares discriminant analysis; SD, standard deviation; Sia, sialic acid; VIP, variable importance in the projection.
