# Supplementary material for: Isomer-Specific Monitoring of Sialylated N-Glycans Reveals Association of α2,3-Linked Sialic Acid Epitope With Behcet’s Disease
Source: Front Mol Biosci. 2021 Nov 23;8:778851. doi: 10.3389/fmolb.2021.778851 (PMC8650305; doi:10.3389/fmolb.2021.778851)

Supplementary Material

**Table S1.** Clinical characteristics of healthy control and Behcet's disease patient groups.

|  | **Healthy Control** | | **Behcet's Disease Patient** | |
| --- | --- | --- | --- | --- |
|  | **Case (n)** | **Mean Age (Range)** | **Case (n)** | **Mean Age (Range)** |
| **Male** | 26 | 48 (21 – 64) | 26 | 49 (30 – 64) |
| **Female** | 21 | 46 (33 – 69) | 21 | 47 (34 – 67) |
| **Total** | 47 | 47 (21 – 69) | 47 | 48 (30 – 67) |

**Table S2.** MRM transitions and their parameters for monitoring of sialylated glycan isomers.

| **Glycan Isomer** | | **Mass (Da)** | **Precursor Ion (*m/z*)** | **Product Ion (*m/z*)** | **RT (min)** | **Collision Energy (eV)** |
| --- | --- | --- | --- | --- | --- | --- |
| 5_4_0_1 ^a^ | 1^b^ | 1931.6876 | 644.9 | 366.1, 274.1 | 9.0 ± 0.5 | 15 |
|  | 2 | 1931.6876 | 644.9 | 366.1, 274.1 | 10.25 ± 0.5 | 15 |
|  | 3 | 1931.6876 | 644.9 | 366.1, 274.1 | 14.25 ± 0.5 | 15 |
|  | 4 | 1931.6876 | 644.9 | 366.1, 274.1 | 15.0 ± 0.5 | 15 |
|  | 5 | 1931.6876 | 644.9 | 366.1, 274.1 | 15.75 ± 0.5 | 15 |
|  | 6 | 1931.6876 | 644.9 | 366.1, 274.1 | 16.45 ± 0.5 | 15 |
| 5_4_1_1 | 1 | 2077.7455 | 1039.9 | 366.1, 274.1 | 16.60 ± 0.5 | 15 |
|  | 2 | 2077.7455 | 1039.9 | 366.1, 274.1 | 17.25 ± 0.25 | 15 |
|  | 3 | 2077.7455 | 1039.9 | 366.1, 274.1 | 17.75 ± 0.25 | 15 |
| 5_4_0_2 | 1 | 2222.7830 | 741.9 | 366.1, 274.1 | 14.75 ± 1.0 | 15 |
|  | 2 | 2222.7830 | 741.9 | 366.1, 274.1 | 23.75 ± 2.75 | 15 |

^a^ 4-digit glycan annotation (Glycan composition: #Hex_#HexNAc_#Fuc_#NeuAc)

^b^ the eluting order of isomers on PGC LC

**Table S3.** List of normalized absolute peak intensities (NAPIs) and statistical analysis results of glycan composition by MALDI-MS analysis. (average ± SD, n=3)

| **Glycan Composition^a^** | **Glycan Type** | **Healthy Control** | | | | **BD Patient** | | | ***p*-Value** |
| --- | --- | --- | --- | --- | --- | --- | --- | --- | --- |
|  |  | **Average** | **±** | **SD** | **Average** | | **±** | **SD** |  |
| **4_2_0_0** | Neutral | 0.000 | ± | 0.000 | 0.002 | | ± | 0.011 | 3.148.E-01 |
| **3_3_0_0** | Neutral | 0.014 | ± | 0.050 | 0.004 | | ± | 0.025 | 1.874.E-01 |
| **5_2_0_0** | Neutral | 5.404 | ± | 2.139 | 4.779 | | ± | 1.993 | 1.444.E-01 |
| **3_3_1_0** | Neutral | 0.257 | ± | 0.114 | 0.310 | | ± | 0.067 | 7.817.E-03 |
| **4_3_0_0** | Neutral | 0.109 | ± | 0.127 | 0.091 | | ± | 0.109 | 4.405.E-01 |
| **3_4_0_0** | Neutral | 0.755 | ± | 0.407 | 0.826 | | ± | 0.477 | 4.415.E-01 |
| **6_2_0_0** | Neutral | 4.454 | ± | 2.092 | 4.776 | | ± | 2.371 | 4.838.E-01 |
| **4_3_1_0** | Neutral | 0.153 | ± | 0.133 | 0.215 | | ± | 0.073 | 8.182.E-03 |
| **5_3_0_0** | Neutral | 0.394 | ± | 0.248 | 0.367 | | ± | 0.224 | 5.547.E-01 |
| **3_4_1_0** | Neutral | 24.575 | ± | 5.732 | 27.868 | | ± | 8.588 | 3.008.E-02 |
| **4_4_0_0** | Neutral | 1.938 | ± | 0.775 | 1.782 | | ± | 0.888 | 3.639.E-01 |
| **3_5_0_0** | Neutral | 0.496 | ± | 0.239 | 0.679 | | ± | 0.401 | 8.194.E-03 |
| **7_2_0_0** | Neutral | 1.114 | ± | 0.471 | 1.105 | | ± | 0.586 | 9.360.E-01 |
| **5_3_1_0** | Neutral | 0.071 | ± | 0.108 | 0.062 | | ± | 0.094 | 6.516.E-01 |
| **6_3_0_0** | Neutral | 0.207 | ± | 0.139 | 0.202 | | ± | 0.104 | 8.177.E-01 |
| **4_4_1_0** | Neutral | 33.044 | ± | 4.121 | 31.076 | | ± | 3.883 | 1.860.E-02 |
| **5_4_0_0** | Neutral | 1.635 | ± | 0.448 | 1.357 | | ± | 0.671 | 1.954.E-02 |
| **3_5_1_0** | Neutral | 2.734 | ± | 0.888 | 3.501 | | ± | 1.026 | 1.838.E-04 |
| **4_5_0_0** | Neutral | 0.536 | ± | 0.170 | 0.627 | | ± | 0.230 | 3.124.E-02 |
| **8_2_0_0** | Neutral | 1.878 | ± | 0.771 | 1.760 | | ± | 0.894 | 4.928.E-01 |
| **5_4_1_0** | Neutral | 14.285 | ± | 4.016 | 12.145 | | ± | 4.338 | 1.436.E-02 |
| **6_4_0_0** | Neutral | 0.068 | ± | 0.123 | 0.040 | | ± | 0.064 | 1.646.E-01 |
| **3_5_2_0** | Neutral | 0.017 | ± | 0.055 | 0.041 | | ± | 0.238 | 4.900.E-01 |
| **4_5_1_0** | Neutral | 3.302 | ± | 0.760 | 3.894 | | ± | 1.018 | 1.776.E-03 |
| **5_5_0_0** | Neutral | 0.115 | ± | 0.105 | 0.133 | | ± | 0.083 | 4.023.E-01 |
| **9_2_0_0** | Neutral | 1.548 | ± | 0.713 | 1.431 | | ± | 0.723 | 4.351.E-01 |
| **5_5_1_0** | Neutral | 0.893 | ± | 0.283 | 0.904 | | ± | 0.397 | 8.803.E-01 |
| **10_2_0_0** | Neutral | 0.003 | ± | 0.016 | 0.021 | | ± | 0.045 | 1.121.E-02 |
| **5_4_0_1** | Acidic | 74.342 | ± | 4.701 | 81.122 | | ± | 3.080 | 7.463E-13 |
| **5_4_1_1** | Acidic | 10.099 | ± | 2.539 | 7.215 | | ± | 3.007 | 2.423E-06 |
| **5_4_0_2** | Acidic | 13.570 | ± | 3.657 | 9.619 | | ± | 1.978 | 2.554E-09 |
| **6_5_0_1** | Acidic | 1.988 | ± | 0.722 | 2.044 | | ± | 0.713 | 6.459E-01 |

^a^ 4-digit glycan annotation (Glycan composition: #Hex_#HexNAc_#Fuc_#NeuAc)

**Table S4.** The absolute abundances of sialylated glycan isomers by PGC-LC/MRM-MS analysis. (average ± SD, n=2)

| **Sample^a^** | **5401_1^b^** | | | **5401_2** | | | **5401_3** | | | **5401_4** | | | **5401_5** | | | **5401_6** | | | **5411_1** | | | **5411_2** | | | **5411_3** | | | **5402_1** | | | **5402_2** | | |
| --- | --- | --- | --- | --- | --- | --- | --- | --- | --- | --- | --- | --- | --- | --- | --- | --- | --- | --- | --- | --- | --- | --- | --- | --- | --- | --- | --- | --- | --- | --- | --- | --- | --- |
| **NM-5** | 27.2 | ± | 8.7 | 167.0 | ± | 18.1 | 2829.1 | ± | 18.0 | 5264.5 | ± | 72.8 | 5512.9 | ± | 121.6 | 10256.0 | ± | 26.4 | 736.0 | ± | 43.8 | 615.7 | ± | 54.6 | 1612.8 | ± | 18.2 | 84.5 | ± | 10.3 | 29578.8 | ± | 426.5 |
| **NM-11** | 11.7 | ± | 2.2 | 64.2 | ± | 3.4 | 2393.6 | ± | 74.7 | 4796.5 | ± | 9.3 | 4655.0 | ± | 55.5 | 9378.3 | ± | 116.0 | 678.0 | ± | 0.3 | 521.5 | ± | 44.6 | 1648.2 | ± | 36.2 | 0.0 | ± | 0.0 | 25840.3 | ± | 373.5 |
| **NM-13** | 26.7 | ± | 2.3 | 113.9 | ± | 7.2 | 2173.4 | ± | 5.8 | 4152.0 | ± | 70.0 | 4252.9 | ± | 33.8 | 8002.5 | ± | 76.4 | 675.6 | ± | 27.8 | 416.0 | ± | 13.5 | 1601.7 | ± | 4.7 | 77.8 | ± | 0.0 | 23381.2 | ± | 397.6 |
| **NM-15** | 21.2 | ± | 4.8 | 79.5 | ± | 6.7 | 2532.1 | ± | 58.9 | 4671.0 | ± | 117.7 | 4910.8 | ± | 141.5 | 9230.3 | ± | 36.8 | 829.6 | ± | 64.2 | 500.6 | ± | 5.4 | 1966.2 | ± | 35.0 | 76.6 | ± | 0.0 | 29683.2 | ± | 483.5 |
| **NM-18** | 30.5 | ± | 1.3 | 199.0 | ± | 0.3 | 2748.1 | ± | 112.9 | 5273.2 | ± | 13.9 | 5351.2 | ± | 127.4 | 10501.6 | ± | 121.1 | 677.4 | ± | 75.3 | 422.8 | ± | 7.2 | 1844.9 | ± | 0.6 | 145.3 | ± | 9.2 | 33077.4 | ± | 162.7 |
| **NM-19** | 31.7 | ± | 3.7 | 120.7 | ± | 4.0 | 2340.0 | ± | 56.5 | 4727.5 | ± | 2.7 | 4632.0 | ± | 27.3 | 9223.6 | ± | 109.9 | 899.1 | ± | 39.8 | 611.6 | ± | 13.4 | 2212.1 | ± | 82.9 | 121.4 | ± | 4.9 | 26443.1 | ± | 189.5 |
| **NM-21** | 25.6 | ± | 2.9 | 87.0 | ± | 4.8 | 1998.6 | ± | 19.9 | 3873.3 | ± | 9.9 | 3885.5 | ± | 7.9 | 7559.6 | ± | 29.6 | 520.2 | ± | 38.2 | 384.1 | ± | 2.2 | 1227.8 | ± | 18.1 | 89.0 | ± | 7.1 | 22392.3 | ± | 202.1 |
| **NM-22** | 24.5 | ± | 2.2 | 111.1 | ± | 12.8 | 1847.6 | ± | 3.7 | 3775.7 | ± | 54.9 | 3579.1 | ± | 128.6 | 7320.4 | ± | 39.5 | 708.9 | ± | 38.1 | 515.1 | ± | 19.0 | 1763.6 | ± | 48.6 | 97.5 | ± | 14.8 | 21536.9 | ± | 207.0 |
| **NM-23** | 40.4 | ± | 8.5 | 91.4 | ± | 4.7 | 2650.8 | ± | 68.7 | 4298.9 | ± | 14.3 | 5056.8 | ± | 34.2 | 8510.2 | ± | 136.1 | 664.8 | ± | 66.6 | 586.6 | ± | 30.6 | 1476.9 | ± | 20.9 | 0.0 | ± | 0.0 | 17472.4 | ± | 165.9 |
| **NM-24** | 38.0 | ± | 1.6 | 136.9 | ± | 2.0 | 2088.1 | ± | 60.2 | 3853.1 | ± | 28.0 | 4201.9 | ± | 53.5 | 7802.0 | ± | 47.6 | 615.0 | ± | 20.4 | 462.3 | ± | 45.3 | 1497.0 | ± | 26.8 | 167.7 | ± | 30.1 | 27126.3 | ± | 207.1 |
| **NM-25** | 45.2 | ± | 9.3 | 216.2 | ± | 10.9 | 3612.5 | ± | 120.0 | 6898.5 | ± | 74.8 | 7163.1 | ± | 43.8 | 13240.3 | ± | 134.7 | 885.8 | ± | 36.5 | 773.4 | ± | 0.2 | 1903.5 | ± | 7.8 | 138.1 | ± | 1.8 | 39690.8 | ± | 181.0 |
| **NM-26** | 40.4 | ± | 3.2 | 183.3 | ± | 11.0 | 2904.2 | ± | 1.0 | 5486.5 | ± | 72.1 | 5473.3 | ± | 8.6 | 10601.0 | ± | 2.4 | 656.5 | ± | 19.4 | 451.3 | ± | 11.6 | 1670.4 | ± | 15.3 | 158.2 | ± | 6.0 | 32451.0 | ± | 78.4 |
| **NM-29** | 43.5 | ± | 5.8 | 205.5 | ± | 9.8 | 2820.7 | ± | 18.6 | 5075.3 | ± | 69.7 | 5560.7 | ± | 60.0 | 10088.4 | ± | 47.2 | 617.7 | ± | 20.7 | 442.8 | ± | 15.0 | 1506.6 | ± | 36.4 | 194.6 | ± | 19.7 | 32157.4 | ± | 336.6 |
| **NM-30** | 23.6 | ± | 4.8 | 87.1 | ± | 0.8 | 2403.6 | ± | 38.0 | 4492.4 | ± | 216.0 | 4839.5 | ± | 89.0 | 8564.9 | ± | 35.8 | 615.4 | ± | 26.1 | 466.0 | ± | 37.1 | 1393.7 | ± | 1.4 | 86.4 | ± | 11.2 | 27502.6 | ± | 139.0 |
| **NM-31** | 59.7 | ± | 9.1 | 197.4 | ± | 22.5 | 3229.1 | ± | 28.1 | 5823.3 | ± | 55.6 | 6053.4 | ± | 28.2 | 11317.0 | ± | 506.0 | 853.3 | ± | 20.2 | 810.1 | ± | 23.8 | 1907.3 | ± | 31.0 | 180.7 | ± | 28.2 | 36025.7 | ± | 995.3 |
| **NM-32** | 28.0 | ± | 0.4 | 96.6 | ± | 11.7 | 2280.9 | ± | 15.1 | 4954.1 | ± | 119.0 | 4226.0 | ± | 1.6 | 9683.5 | ± | 343.7 | 474.5 | ± | 22.6 | 366.7 | ± | 4.7 | 1156.4 | ± | 12.9 | 91.0 | ± | 3.2 | 25803.3 | ± | 502.0 |
| **NM-33** | 52.5 | ± | 4.5 | 151.9 | ± | 7.8 | 3225.3 | ± | 4.9 | 5470.3 | ± | 140.2 | 6120.0 | ± | 46.1 | 11422.9 | ± | 117.1 | 923.4 | ± | 15.2 | 803.6 | ± | 40.8 | 1991.6 | ± | 40.8 | 129.7 | ± | 13.8 | 36804.6 | ± | 326.3 |
| **NM-34** | 27.1 | ± | 0.2 | 78.4 | ± | 3.9 | 2182.4 | ± | 23.9 | 4164.0 | ± | 39.6 | 4084.1 | ± | 175.3 | 8308.3 | ± | 210.2 | 605.5 | ± | 9.9 | 484.7 | ± | 17.1 | 1509.4 | ± | 8.6 | 77.0 | ± | 10.1 | 25166.3 | ± | 67.5 |
| **NM-36** | 49.2 | ± | 1.3 | 133.8 | ± | 3.3 | 3233.6 | ± | 63.9 | 5529.1 | ± | 19.8 | 5936.6 | ± | 154.8 | 10778.3 | ± | 63.6 | 919.1 | ± | 36.0 | 790.9 | ± | 11.2 | 2210.1 | ± | 31.3 | 140.9 | ± | 11.1 | 36006.9 | ± | 46.9 |
| **NM-37** | 41.4 | ± | 10.2 | 147.5 | ± | 4.2 | 2802.5 | ± | 81.5 | 5702.0 | ± | 12.4 | 5127.5 | ± | 59.3 | 11216.2 | ± | 46.5 | 692.9 | ± | 13.6 | 514.0 | ± | 11.0 | 1592.0 | ± | 3.4 | 139.1 | ± | 11.7 | 31490.8 | ± | 155.3 |
| **NM-38** | 196.2 | ± | 2.0 | 466.5 | ± | 6.2 | 2246.4 | ± | 25.1 | 4556.6 | ± | 28.2 | 4330.0 | ± | 59.8 | 9224.7 | ± | 38.6 | 616.5 | ± | 1.7 | 419.0 | ± | 31.8 | 1477.6 | ± | 29.0 | 725.4 | ± | 24.7 | 28972.6 | ± | 2.0 |
| **NM-39** | 0.0 | ± | 0.0 | 13.9 | ± | 1.7 | 564.8 | ± | 26.1 | 1242.1 | ± | 58.4 | 1125.7 | ± | 13.8 | 2518.9 | ± | 99.0 | 221.9 | ± | 2.6 | 140.1 | ± | 15.0 | 551.0 | ± | 7.4 | 0.0 | ± | 0.0 | 8543.9 | ± | 234.5 |
| **NM-40** | 344.4 | ± | 4.7 | 973.5 | ± | 38.4 | 2842.6 | ± | 96.7 | 6601.7 | ± | 225.2 | 5194.6 | ± | 357.2 | 13192.0 | ± | 234.1 | 1066.4 | ± | 19.0 | 791.2 | ± | 33.9 | 2324.2 | ± | 63.8 | 1223.0 | ± | 25.9 | 37153.4 | ± | 851.3 |
| **NM-45** | 207.6 | ± | 1.1 | 488.0 | ± | 10.7 | 2418.7 | ± | 17.6 | 4442.5 | ± | 92.6 | 4444.9 | ± | 93.1 | 8823.1 | ± | 60.8 | 914.6 | ± | 2.5 | 568.4 | ± | 28.2 | 2209.8 | ± | 71.5 | 817.6 | ± | 21.2 | 32544.0 | ± | 0.7 |
| **NM-47** | 20.7 | ± | 2.2 | 68.9 | ± | 6.2 | 1228.1 | ± | 10.6 | 3115.0 | ± | 104.3 | 2465.7 | ± | 3.5 | 6386.5 | ± | 37.8 | 463.5 | ± | 19.2 | 257.9 | ± | 9.5 | 1214.5 | ± | 2.0 | 147.2 | ± | 14.9 | 25220.0 | ± | 445.4 |
| **NM-53** | 144.1 | ± | 3.4 | 428.3 | ± | 4.9 | 1858.7 | ± | 20.3 | 4225.6 | ± | 115.5 | 3577.0 | ± | 88.7 | 8721.7 | ± | 55.6 | 721.3 | ± | 9.2 | 382.3 | ± | 2.6 | 1721.6 | ± | 71.0 | 752.1 | ± | 43.8 | 32027.8 | ± | 206.6 |
| **NF-2** | 724.8 | ± | 37.0 | 1861.7 | ± | 36.7 | 3328.9 | ± | 81.2 | 7250.3 | ± | 134.9 | 6333.2 | ± | 106.8 | 14330.9 | ± | 271.5 | 1457.4 | ± | 68.6 | 584.1 | ± | 15.9 | 3761.2 | ± | 29.3 | 2946.5 | ± | 126.7 | 55336.0 | ± | 296.9 |
| **NF-3** | 561.2 | ± | 19.5 | 1796.9 | ± | 30.5 | 2645.6 | ± | 48.6 | 6817.3 | ± | 93.1 | 4935.4 | ± | 448.0 | 13146.8 | ± | 193.8 | 1434.8 | ± | 25.4 | 718.8 | ± | 37.1 | 3527.9 | ± | 10.5 | 2182.7 | ± | 1107.5 | 51259.5 | ± | 116.6 |
| **NF-4** | 401.9 | ± | 9.9 | 988.2 | ± | 10.7 | 2099.2 | ± | 46.0 | 4780.0 | ± | 45.9 | 3796.0 | ± | 564.1 | 9698.7 | ± | 113.6 | 971.3 | ± | 28.0 | 427.8 | ± | 9.0 | 2639.1 | ± | 29.3 | 1750.9 | ± | 35.7 | 36818.0 | ± | 23.6 |
| **NF-17** | 280.8 | ± | 7.0 | 928.9 | ± | 7.8 | 1954.6 | ± | 90.1 | 5203.0 | ± | 3.3 | 3847.3 | ± | 87.4 | 10451.0 | ± | 140.1 | 1096.3 | ± | 3.6 | 514.3 | ± | 15.3 | 2919.2 | ± | 72.7 | 1335.5 | ± | 18.8 | 34191.9 | ± | 245.5 |
| **NF-18** | 316.3 | ± | 13.0 | 1087.2 | ± | 6.3 | 2105.7 | ± | 32.5 | 6148.3 | ± | 119.3 | 4212.9 | ± | 62.2 | 12151.0 | ± | 128.1 | 1146.0 | ± | 1.5 | 529.5 | ± | 0.1 | 2884.6 | ± | 56.9 | 1208.9 | ± | 539.4 | 38059.8 | ± | 29.0 |
| **NF-19** | 162.1 | ± | 1.0 | 439.9 | ± | 14.9 | 1810.4 | ± | 12.0 | 4307.5 | ± | 135.2 | 3586.6 | ± | 16.0 | 8473.2 | ± | 75.3 | 892.2 | ± | 32.2 | 281.4 | ± | 24.3 | 2538.1 | ± | 9.4 | 827.2 | ± | 18.8 | 33119.5 | ± | 338.2 |
| **NF-20** | 170.5 | ± | 9.9 | 413.2 | ± | 8.8 | 2965.2 | ± | 49.3 | 6114.8 | ± | 148.7 | 5809.2 | ± | 55.0 | 11993.4 | ± | 308.8 | 827.6 | ± | 39.7 | 569.6 | ± | 24.3 | 1952.9 | ± | 8.3 | 464.5 | ± | 7.8 | 27542.8 | ± | 145.5 |
| **NF-21** | 276.3 | ± | 25.7 | 769.8 | ± | 5.4 | 2208.2 | ± | 6.5 | 5550.2 | ± | 77.7 | 4503.9 | ± | 65.8 | 11094.6 | ± | 109.7 | 843.5 | ± | 4.4 | 440.1 | ± | 19.5 | 2082.6 | ± | 3.0 | 1493.1 | ± | 37.8 | 42596.9 | ± | 154.2 |
| **NF-22** | 161.9 | ± | 10.2 | 487.7 | ± | 10.3 | 1949.7 | ± | 19.3 | 4277.0 | ± | 37.1 | 3814.6 | ± | 68.3 | 8627.0 | ± | 187.8 | 920.3 | ± | 1.9 | 394.3 | ± | 29.7 | 2375.6 | ± | 96.5 | 818.2 | ± | 27.8 | 32956.1 | ± | 185.2 |
| **NF-26** | 213.5 | ± | 22.8 | 456.1 | ± | 0.5 | 3106.6 | ± | 162.2 | 5604.7 | ± | 209.9 | 6032.9 | ± | 17.2 | 11259.9 | ± | 171.2 | 1202.2 | ± | 33.5 | 716.7 | ± | 67.2 | 2898.1 | ± | 3.1 | 487.8 | ± | 7.8 | 26996.3 | ± | 382.1 |
| **NF-28** | 287.8 | ± | 14.7 | 729.5 | ± | 18.1 | 1910.1 | ± | 12.5 | 4679.4 | ± | 345.2 | 5007.4 | ± | 227.2 | 10545.4 | ± | 151.8 | 1101.0 | ± | 96.2 | 573.0 | ± | 4.9 | 2836.2 | ± | 2.9 | 1283.6 | ± | 22.3 | 42814.7 | ± | 1491.2 |
| **NF-29** | 497.5 | ± | 1.6 | 1338.5 | ± | 14.4 | 2417.5 | ± | 33.2 | 5108.8 | ± | 45.4 | 4911.7 | ± | 29.5 | 9862.8 | ± | 152.9 | 810.5 | ± | 1.3 | 539.4 | ± | 37.1 | 1917.0 | ± | 32.4 | 1984.9 | ± | 106.8 | 36124.5 | ± | 139.9 |
| **NF-31** | 221.1 | ± | 16.4 | 585.3 | ± | 32.9 | 2318.5 | ± | 13.9 | 4771.5 | ± | 30.3 | 4474.7 | ± | 44.5 | 9897.1 | ± | 137.4 | 727.4 | ± | 2.1 | 402.6 | ± | 5.8 | 1783.5 | ± | 28.1 | 998.3 | ± | 26.0 | 39573.6 | ± | 356.9 |
| **NF-35** | 227.9 | ± | 12.8 | 653.3 | ± | 20.0 | 1777.8 | ± | 72.2 | 4350.8 | ± | 60.5 | 3484.5 | ± | 4.0 | 8888.6 | ± | 240.6 | 847.9 | ± | 6.8 | 498.7 | ± | 34.6 | 2135.8 | ± | 40.9 | 1117.1 | ± | 12.6 | 32118.1 | ± | 36.6 |
| **NF-37** | 446.9 | ± | 1.6 | 980.0 | ± | 40.5 | 3615.9 | ± | 178.7 | 6912.0 | ± | 184.2 | 7320.7 | ± | 83.5 | 13439.1 | ± | 119.1 | 1012.9 | ± | 24.4 | 906.5 | ± | 26.2 | 2042.9 | ± | 70.0 | 707.1 | ± | 15.7 | 24679.6 | ± | 281.8 |
| **NF-38** | 341.7 | ± | 16.8 | 802.4 | ± | 7.0 | 2138.8 | ± | 46.0 | 4341.8 | ± | 76.0 | 4219.5 | ± | 17.1 | 8415.5 | ± | 33.8 | 723.9 | ± | 2.4 | 455.6 | ± | 9.5 | 1924.1 | ± | 50.3 | 1636.3 | ± | 10.4 | 38753.3 | ± | 110.0 |
| **NF-39** | 554.1 | ± | 11.4 | 1509.8 | ± | 22.6 | 2776.2 | ± | 42.5 | 6314.2 | ± | 252.6 | 5404.0 | ± | 171.6 | 12215.0 | ± | 155.9 | 933.3 | ± | 14.3 | 545.9 | ± | 14.6 | 2289.1 | ± | 100.9 | 2558.9 | ± | 25.9 | 46808.7 | ± | 780.6 |
| **NF-46** | 721.0 | ± | 18.2 | 1443.0 | ± | 71.4 | 4736.3 | ± | 9.8 | 7368.8 | ± | 62.2 | 9188.6 | ± | 26.0 | 13897.2 | ± | 182.2 | 794.1 | ± | 26.7 | 781.9 | ± | 22.5 | 1620.4 | ± | 49.0 | 1374.4 | ± | 51.8 | 34354.3 | ± | 123.7 |
| **NF-49** | 503.3 | ± | 6.9 | 1209.4 | ± | 3.9 | 4323.9 | ± | 5.9 | 8012.6 | ± | 36.9 | 8512.8 | ± | 137.1 | 15304.1 | ± | 209.1 | 829.9 | ± | 20.6 | 831.3 | ± | 15.1 | 1803.8 | ± | 13.3 | 827.0 | ± | 44.4 | 28588.8 | ± | 104.9 |
| **NF-50** | 347.1 | ± | 2.4 | 974.8 | ± | 42.9 | 2255.5 | ± | 9.6 | 5618.2 | ± | 45.1 | 4557.2 | ± | 20.0 | 10540.1 | ± | 11.7 | 562.7 | ± | 5.4 | 293.5 | ± | 4.2 | 1447.3 | ± | 2.1 | 1713.6 | ± | 100.9 | 40461.6 | ± | 280.0 |
| **NF-55** | 338.0 | ± | 21.0 | 917.7 | ± | 0.2 | 2281.3 | ± | 48.6 | 5890.8 | ± | 86.2 | 4558.2 | ± | 82.3 | 11099.7 | ± | 139.6 | 769.3 | ± | 5.5 | 482.5 | ± | 29.2 | 1882.6 | ± | 62.3 | 1566.2 | ± | 7.5 | 40419.0 | ± | 177.3 |
| **PM-1** | 4049.9 | ± | 184.4 | 10445.9 | ± | 231.8 | 4164.0 | ± | 186.4 | 8839.6 | ± | 352.9 | 7736.3 | ± | 269.4 | 17589.1 | ± | 469.0 | 1297.7 | ± | 5.9 | 660.7 | ± | 68.5 | 3082.0 | ± | 181.7 | 15674.3 | ± | 424.4 | 72325.4 | ± | 2263.8 |
| **PM-3** | 1472.0 | ± | 9.6 | 4441.6 | ± | 57.2 | 1972.2 | ± | 24.3 | 4973.0 | ± | 79.2 | 3277.9 | ± | 22.2 | 10036.1 | ± | 181.8 | 534.1 | ± | 18.7 | 317.3 | ± | 8.6 | 1274.5 | ± | 23.2 | 6017.8 | ± | 42.9 | 33870.1 | ± | 1273.5 |
| **PM-6** | 2130.1 | ± | 36.7 | 6084.2 | ± | 248.8 | 2665.8 | ± | 34.8 | 6181.4 | ± | 33.0 | 5405.8 | ± | 27.0 | 12464.4 | ± | 132.9 | 994.6 | ± | 18.3 | 705.5 | ± | 11.9 | 2229.2 | ± | 38.5 | 8139.8 | ± | 12.5 | 45263.6 | ± | 99.4 |
| **PM-3** | 1325.4 | ± | 14.7 | 3617.6 | ± | 80.1 | 2077.7 | ± | 6.0 | 4414.5 | ± | 31.0 | 4010.1 | ± | 50.5 | 9080.3 | ± | 211.4 | 836.2 | ± | 22.6 | 632.5 | ± | 41.0 | 2005.5 | ± | 10.0 | 6380.6 | ± | 83.8 | 38315.8 | ± | 466.7 |
| **PM-6** | 1376.0 | ± | 20.9 | 3373.9 | ± | 65.9 | 2214.3 | ± | 74.0 | 4092.3 | ± | 81.9 | 4192.8 | ± | 1.3 | 8570.8 | ± | 321.7 | 901.0 | ± | 13.6 | 600.6 | ± | 26.9 | 2177.3 | ± | 113.6 | 6496.3 | ± | 158.6 | 38959.6 | ± | 1077.3 |
| **PM-7** | 1119.9 | ± | 10.7 | 3020.0 | ± | 3.2 | 1906.0 | ± | 38.1 | 4061.8 | ± | 44.7 | 3813.6 | ± | 36.4 | 8210.0 | ± | 301.3 | 592.3 | ± | 16.5 | 379.1 | ± | 8.1 | 1511.4 | ± | 25.2 | 5580.2 | ± | 76.9 | 33913.7 | ± | 214.8 |
| **PM-8** | 1108.4 | ± | 1.8 | 3111.2 | ± | 75.8 | 1981.5 | ± | 14.5 | 4152.2 | ± | 145.7 | 3810.2 | ± | 24.0 | 8555.9 | ± | 121.7 | 1401.0 | ± | 7.9 | 969.1 | ± | 3.8 | 3493.0 | ± | 3.6 | 5231.2 | ± | 82.3 | 34045.2 | ± | 511.3 |
| **PM-11** | 1396.4 | ± | 34.5 | 3602.2 | ± | 9.5 | 2406.2 | ± | 84.4 | 4756.3 | ± | 31.9 | 4346.5 | ± | 543.9 | 9428.6 | ± | 71.2 | 741.0 | ± | 40.4 | 610.5 | ± | 30.6 | 1660.0 | ± | 11.3 | 6552.2 | ± | 262.8 | 40659.9 | ± | 530.7 |
| **PM-13** | 970.8 | ± | 12.6 | 2484.7 | ± | 24.9 | 2364.4 | ± | 17.8 | 4315.5 | ± | 98.1 | 4549.0 | ± | 87.9 | 8950.6 | ± | 120.0 | 638.1 | ± | 25.4 | 456.7 | ± | 22.2 | 1498.4 | ± | 38.6 | 4144.1 | ± | 137.6 | 33454.4 | ± | 232.6 |
| **PM-14** | 475.6 | ± | 3.0 | 1268.7 | ± | 11.2 | 1926.6 | ± | 132.0 | 3756.8 | ± | 256.7 | 3745.8 | ± | 148.4 | 7942.1 | ± | 416.1 | 593.5 | ± | 3.1 | 353.2 | ± | 4.4 | 1538.5 | ± | 95.3 | 2537.1 | ± | 12.8 | 31013.6 | ± | 981.4 |
| **PM-15** | 364.1 | ± | 20.5 | 849.9 | ± | 36.3 | 1759.8 | ± | 19.9 | 3343.0 | ± | 32.9 | 3396.7 | ± | 24.5 | 7034.5 | ± | 58.5 | 507.2 | ± | 17.8 | 263.2 | ± | 2.7 | 1400.0 | ± | 70.0 | 1863.4 | ± | 35.1 | 28561.8 | ± | 179.8 |
| **PM-16** | 681.0 | ± | 16.7 | 1959.7 | ± | 25.5 | 1977.9 | ± | 17.1 | 4016.3 | ± | 26.5 | 3900.6 | ± | 97.0 | 8372.2 | ± | 38.0 | 754.1 | ± | 25.7 | 414.8 | ± | 4.9 | 1884.2 | ± | 1.8 | 3443.4 | ± | 50.3 | 32111.6 | ± | 59.3 |
| **PM-17** | 859.4 | ± | 33.3 | 2469.0 | ± | 29.8 | 2297.7 | ± | 7.6 | 4944.4 | ± | 35.8 | 4433.1 | ± | 79.0 | 9835.5 | ± | 158.1 | 610.9 | ± | 54.7 | 484.8 | ± | 24.4 | 1517.4 | ± | 30.4 | 3952.9 | ± | 70.2 | 36513.7 | ± | 308.0 |
| **PM-18** | 720.6 | ± | 8.4 | 1917.2 | ± | 62.9 | 1996.8 | ± | 61.6 | 3666.9 | ± | 84.7 | 3816.1 | ± | 38.4 | 7454.1 | ± | 171.1 | 595.9 | ± | 8.2 | 498.1 | ± | 31.5 | 1575.7 | ± | 13.3 | 3453.3 | ± | 66.2 | 29863.9 | ± | 80.1 |
| **PM-20** | 1079.6 | ± | 40.5 | 2859.1 | ± | 45.3 | 2936.7 | ± | 14.6 | 5690.3 | ± | 7.9 | 5864.4 | ± | 56.1 | 11484.9 | ± | 193.9 | 1287.6 | ± | 22.5 | 834.7 | ± | 5.3 | 3226.8 | ± | 76.3 | 5275.7 | ± | 148.9 | 47253.5 | ± | 140.6 |
| **PM-21** | 244.4 | ± | 14.4 | 625.9 | ± | 17.5 | 1600.5 | ± | 92.3 | 3172.8 | ± | 100.2 | 3145.5 | ± | 22.9 | 6409.4 | ± | 88.6 | 521.1 | ± | 4.8 | 333.6 | ± | 35.3 | 1426.0 | ± | 10.1 | 1307.9 | ± | 14.7 | 24171.2 | ± | 217.5 |
| **PM-22** | 270.9 | ± | 9.2 | 768.3 | ± | 21.2 | 1890.6 | ± | 15.4 | 4021.8 | ± | 156.6 | 3852.0 | ± | 72.3 | 8174.6 | ± | 173.8 | 1052.5 | ± | 38.5 | 448.8 | ± | 6.5 | 2840.7 | ± | 4.7 | 1545.5 | ± | 19.6 | 30195.7 | ± | 220.6 |
| **PM-24** | 529.5 | ± | 8.0 | 1335.7 | ± | 54.1 | 2475.2 | ± | 15.0 | 4271.9 | ± | 82.9 | 4256.8 | ± | 19.7 | 9046.8 | ± | 100.3 | 914.9 | ± | 16.2 | 730.6 | ± | 21.7 | 2185.6 | ± | 31.9 | 1762.1 | ± | 179.3 | 29027.0 | ± | 2953.8 |
| **PM-25** | 574.5 | ± | 2.7 | 1666.4 | ± | 34.1 | 2288.9 | ± | 39.0 | 4982.2 | ± | 111.7 | 4661.7 | ± | 167.0 | 10484.0 | ± | 319.5 | 831.7 | ± | 0.8 | 513.8 | ± | 38.1 | 2059.2 | ± | 141.6 | 1968.6 | ± | 103.9 | 27408.9 | ± | 1429.2 |
| **PM-27** | 577.7 | ± | 0.7 | 1667.6 | ± | 50.4 | 2192.1 | ± | 50.2 | 4689.9 | ± | 70.1 | 4253.6 | ± | 7.4 | 8929.2 | ± | 32.3 | 575.9 | ± | 19.1 | 312.5 | ± | 21.6 | 1447.5 | ± | 18.5 | 2477.3 | ± | 74.6 | 33212.6 | ± | 122.0 |
| **PM-32** | 345.7 | ± | 16.9 | 763.0 | ± | 4.7 | 2011.3 | ± | 23.6 | 3688.0 | ± | 20.5 | 3918.2 | ± | 63.9 | 7039.8 | ± | 69.6 | 650.1 | ± | 2.3 | 407.6 | ± | 39.0 | 1679.1 | ± | 0.9 | 1407.0 | ± | 57.6 | 30140.1 | ± | 118.1 |
| **PM-33** | 1924.3 | ± | 16.8 | 5144.4 | ± | 22.6 | 2651.6 | ± | 34.5 | 6212.8 | ± | 19.5 | 5675.9 | ± | 57.2 | 11754.0 | ± | 75.2 | 841.9 | ± | 13.9 | 529.0 | ± | 9.9 | 2079.3 | ± | 2.5 | 7676.1 | ± | 525.3 | 45452.2 | ± | 17.7 |
| **PM-34** | 2044.1 | ± | 5.3 | 5275.2 | ± | 50.2 | 2647.1 | ± | 18.3 | 5548.4 | ± | 44.5 | 5375.0 | ± | 37.4 | 10813.0 | ± | 273.5 | 997.0 | ± | 53.0 | 760.2 | ± | 12.7 | 2386.0 | ± | 57.7 | 8063.2 | ± | 330.3 | 43217.4 | ± | 2.2 |
| **PM-37** | 1330.3 | ± | 41.5 | 3134.2 | ± | 9.8 | 2215.9 | ± | 3.2 | 4466.0 | ± | 78.3 | 4605.3 | ± | 74.9 | 8506.2 | ± | 150.9 | 590.9 | ± | 1.1 | 435.2 | ± | 34.1 | 1552.8 | ± | 0.8 | 5315.2 | ± | 0.7 | 37020.8 | ± | 207.9 |
| **PM-38** | 1427.5 | ± | 6.2 | 3918.2 | ± | 58.7 | 2116.7 | ± | 64.9 | 4793.3 | ± | 73.5 | 4338.2 | ± | 130.6 | 9117.4 | ± | 34.7 | 655.0 | ± | 46.6 | 413.8 | ± | 1.6 | 1605.7 | ± | 24.6 | 5495.4 | ± | 36.9 | 33372.4 | ± | 630.8 |
| **PM-39** | 2025.8 | ± | 18.3 | 5020.8 | ± | 120.0 | 2626.9 | ± | 42.0 | 5537.9 | ± | 40.4 | 5335.0 | ± | 160.7 | 10848.0 | ± | 88.3 | 1087.8 | ± | 6.2 | 564.9 | ± | 9.0 | 2754.4 | ± | 31.2 | 7442.4 | ± | 234.5 | 40408.3 | ± | 359.6 |
| **PF-2** | 1947.2 | ± | 49.3 | 6262.8 | ± | 22.0 | 2491.1 | ± | 8.5 | 6629.8 | ± | 156.5 | 4709.3 | ± | 478.9 | 13130.5 | ± | 302.0 | 949.5 | ± | 52.4 | 678.8 | ± | 15.9 | 2293.6 | ± | 7.5 | 7790.0 | ± | 324.0 | 42028.1 | ± | 1054.1 |
| **PF-4** | 1635.6 | ± | 18.4 | 4091.1 | ± | 145.3 | 2605.8 | ± | 102.5 | 5451.6 | ± | 165.3 | 4965.8 | ± | 226.0 | 11200.2 | ± | 345.2 | 852.0 | ± | 0.9 | 590.0 | ± | 0.3 | 1905.6 | ± | 63.2 | 6580.0 | ± | 129.7 | 44987.8 | ± | 1497.3 |
| **PF-5** | 234.9 | ± | 17.4 | 785.2 | ± | 13.1 | 639.0 | ± | 23.2 | 1917.0 | ± | 47.9 | 1305.5 | ± | 15.4 | 3868.6 | ± | 48.8 | 616.0 | ± | 63.1 | 239.0 | ± | 0.5 | 1555.1 | ± | 35.8 | 1117.1 | ± | 53.9 | 11791.1 | ± | 202.0 |
| **PF-7** | 1409.4 | ± | 74.3 | 3812.1 | ± | 163.2 | 1938.7 | ± | 21.9 | 4237.7 | ± | 92.7 | 3907.2 | ± | 14.0 | 8818.0 | ± | 109.3 | 1146.4 | ± | 60.3 | 502.7 | ± | 76.0 | 3009.6 | ± | 33.0 | 5586.4 | ± | 23.2 | 32248.4 | ± | 426.4 |
| **PF-8** | 885.9 | ± | 43.6 | 2637.9 | ± | 72.8 | 2339.6 | ± | 22.4 | 5666.8 | ± | 77.5 | 4174.4 | ± | 583.2 | 11231.2 | ± | 369.5 | 873.3 | ± | 45.9 | 573.3 | ± | 47.4 | 2091.1 | ± | 83.8 | 3519.6 | ± | 110.3 | 36163.7 | ± | 965.2 |
| **PF-1** | 874.4 | ± | 32.7 | 2515.0 | ± | 28.1 | 1577.4 | ± | 44.5 | 3602.9 | ± | 51.2 | 3063.5 | ± | 191.5 | 7699.6 | ± | 93.0 | 648.0 | ± | 13.1 | 405.3 | ± | 5.3 | 1657.2 | ± | 15.0 | 4492.0 | ± | 7.9 | 28739.7 | ± | 570.2 |
| **PF-2** | 1246.9 | ± | 9.9 | 3006.7 | ± | 56.6 | 1952.0 | ± | 19.3 | 3585.9 | ± | 60.2 | 4065.5 | ± | 160.0 | 7601.7 | ± | 163.5 | 731.6 | ± | 4.8 | 412.3 | ± | 19.8 | 1901.2 | ± | 0.6 | 6199.0 | ± | 25.5 | 35471.7 | ± | 706.8 |
| **PF-4** | 914.6 | ± | 14.3 | 2513.7 | ± | 12.8 | 1628.3 | ± | 67.1 | 3567.3 | ± | 33.2 | 2994.1 | ± | 105.9 | 7405.2 | ± | 20.4 | 738.4 | ± | 26.2 | 541.2 | ± | 47.0 | 1725.1 | ± | 5.4 | 4479.3 | ± | 59.3 | 28350.2 | ± | 24.6 |
| **PF-5** | 1315.4 | ± | 1.9 | 3449.2 | ± | 27.7 | 2191.5 | ± | 29.1 | 4631.3 | ± | 84.0 | 4352.5 | ± | 51.7 | 9681.7 | ± | 165.3 | 723.6 | ± | 8.4 | 459.6 | ± | 24.8 | 1835.8 | ± | 9.2 | 6342.4 | ± | 79.7 | 41427.2 | ± | 602.0 |
| **PF-9** | 1286.8 | ± | 20.7 | 3877.5 | ± | 11.9 | 2055.4 | ± | 12.7 | 4996.3 | ± | 6.7 | 4336.5 | ± | 157.1 | 10542.3 | ± | 249.0 | 906.5 | ± | 15.0 | 536.6 | ± | 13.5 | 2319.0 | ± | 21.1 | 6506.8 | ± | 91.6 | 39274.7 | ± | 424.5 |
| **PF-10** | 955.4 | ± | 25.7 | 2412.7 | ± | 22.3 | 2015.4 | ± | 7.4 | 4144.4 | ± | 35.3 | 4057.3 | ± | 53.8 | 8569.7 | ± | 88.8 | 844.6 | ± | 6.4 | 413.9 | ± | 28.8 | 2157.4 | ± | 30.5 | 4813.8 | ± | 43.1 | 35434.1 | ± | 611.5 |
| **PF-12** | 876.1 | ± | 7.6 | 2225.5 | ± | 15.7 | 1734.2 | ± | 22.8 | 3486.6 | ± | 52.8 | 3402.3 | ± | 80.2 | 7351.8 | ± | 101.9 | 883.4 | ± | 3.1 | 815.3 | ± | 14.3 | 1978.7 | ± | 29.0 | 4249.3 | ± | 27.5 | 29302.8 | ± | 269.0 |
| **PF-19** | 212.4 | ± | 7.6 | 629.8 | ± | 0.4 | 1186.8 | ± | 11.7 | 2450.4 | ± | 79.7 | 2319.1 | ± | 32.9 | 5196.2 | ± | 155.3 | 500.1 | ± | 14.0 | 334.2 | ± | 1.2 | 1361.9 | ± | 15.3 | 1313.7 | ± | 31.0 | 19167.2 | ± | 544.0 |
| **PF-23** | 356.3 | ± | 4.5 | 1124.0 | ± | 20.2 | 2192.5 | ± | 38.7 | 5228.5 | ± | 78.8 | 4438.8 | ± | 42.4 | 10747.3 | ± | 3.1 | 1039.8 | ± | 8.8 | 609.2 | ± | 20.3 | 2690.9 | ± | 4.0 | 1788.8 | ± | 2.7 | 34534.3 | ± | 393.9 |
| **PF-26** | 426.8 | ± | 11.8 | 1090.0 | ± | 40.1 | 2216.4 | ± | 18.9 | 4888.0 | ± | 93.7 | 4367.2 | ± | 9.8 | 9363.1 | ± | 124.9 | 1171.4 | ± | 32.5 | 596.9 | ± | 7.1 | 3042.4 | ± | 23.4 | 1808.0 | ± | 47.6 | 35674.8 | ± | 233.8 |
| **PF-28** | 571.8 | ± | 26.0 | 1560.4 | ± | 12.8 | 2694.8 | ± | 60.1 | 6275.5 | ± | 22.1 | 5453.7 | ± | 70.5 | 11861.6 | ± | 208.8 | 1367.7 | ± | 16.4 | 730.9 | ± | 19.6 | 3640.3 | ± | 61.0 | 2185.0 | ± | 25.6 | 40868.9 | ± | 87.4 |
| **PF-29** | 1325.1 | ± | 49.8 | 3248.5 | ± | 19.3 | 3050.7 | ± | 79.2 | 6210.7 | ± | 90.4 | 6233.0 | ± | 107.7 | 12018.0 | ± | 54.9 | 1302.5 | ± | 35.4 | 961.1 | ± | 20.0 | 2947.3 | ± | 35.8 | 4546.7 | ± | 152.4 | 44964.0 | ± | 1179.5 |
| **PF-30** | 725.4 | ± | 0.0 | 1997.7 | ± | 40.7 | 2439.3 | ± | 50.6 | 5415.1 | ± | 56.1 | 4760.2 | ± | 96.0 | 10467.9 | ± | 122.8 | 882.5 | ± | 7.9 | 591.7 | ± | 12.1 | 2221.9 | ± | 70.9 | 2743.1 | ± | 53.5 | 36669.3 | ± | 577.6 |
| **PF-31** | 921.6 | ± | 16.3 | 2289.2 | ± | 16.5 | 2512.9 | ± | 15.7 | 5405.8 | ± | 99.3 | 5224.8 | ± | 16.1 | 10518.7 | ± | 101.3 | 974.7 | ± | 17.2 | 538.1 | ± | 72.1 | 2517.2 | ± | 51.5 | 3293.4 | ± | 20.2 | 39010.3 | ± | 64.6 |
| **PF-35** | 1643.6 | ± | 35.5 | 4490.9 | ± | 34.6 | 2492.9 | ± | 6.1 | 5621.5 | ± | 284.6 | 5293.4 | ± | 10.7 | 11218.1 | ± | 149.7 | 1503.2 | ± | 84.1 | 757.7 | ± | 24.7 | 3724.7 | ± | 2.6 | 6599.1 | ± | 360.9 | 42853.0 | ± | 666.1 |
| **PF-36** | 1847.9 | ± | 1.6 | 5077.5 | ± | 99.8 | 2633.9 | ± | 61.0 | 6039.6 | ± | 27.4 | 5460.7 | ± | 46.3 | 11690.3 | ± | 101.8 | 927.6 | ± | 47.2 | 552.6 | ± | 25.1 | 2392.1 | ± | 16.5 | 6824.3 | ± | 55.5 | 42121.6 | ± | 182.6 |

^a^ NM: healthy control-male; NM: healthy control-female; PM: Behcet's disease patient-male; PF: Behcet's disease patient -female

^b^ Isomer code: glycan composition-elution order

**Figure S1.** Receiver-operating characteristic (ROC) curves of sialylated glycan isomers. Area under the curve (AUC) values with 95% confidence internal (CI) are displayed at the bottom of the plot and sensitivity and specificity are shown at the top of the plot. (Isomer code: glycan composition-elution order)


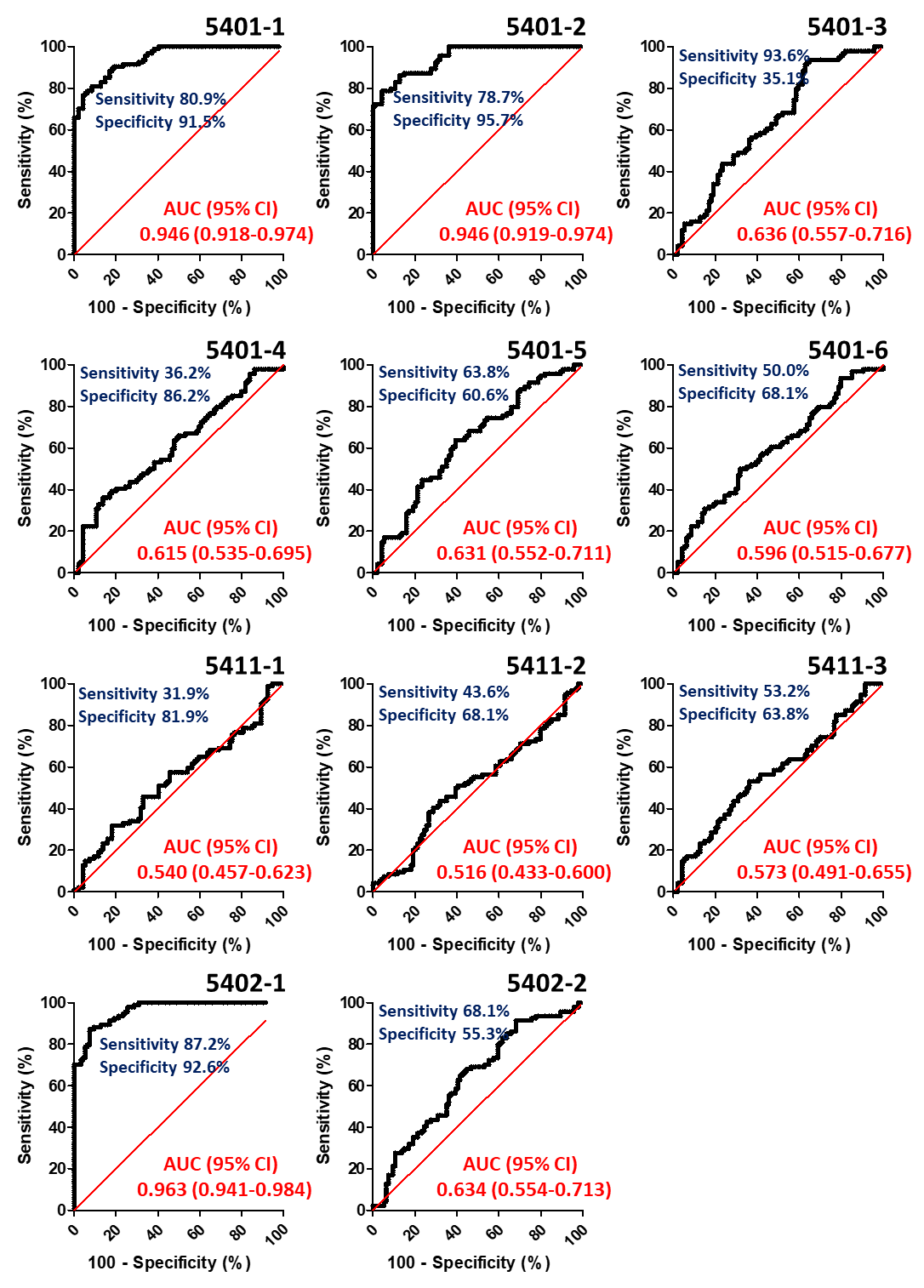


**Figure S2.** Comparison of healthy control group and Behcet’s disease patient group using isomer quantitation of each composition. (A) partial least-squares discriminant analysis (PLS-DA) score plot and (B) variable importance in projection (VIP) score plot derived from PLS-DA analysis. The blue dots are healthy control and red dots are behcet's disease patient, respectively. Colored ellipses represent 95 % confidence intervals (CI).

**
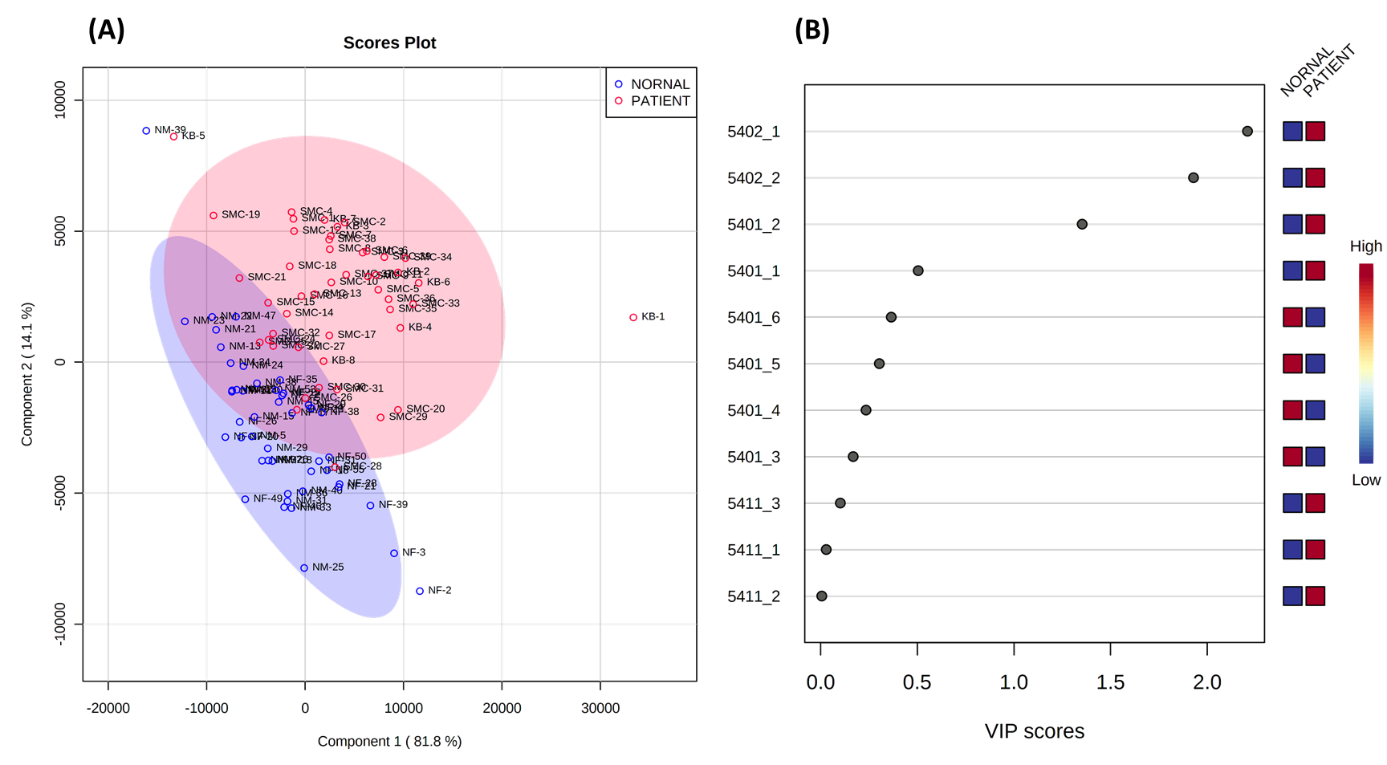
**

**Figure S3.** Comparison of retention time on PGC-LC between serum sialylated glycans and glycan standards corresponding to (A) α2,6 monosialylated biantennary glycan (left arm) and (B) α2,6 disialylated biantennary glycan (left and right arm). Glycan schematic symbols: green circle, mannose; yellow circle, galactose; blue square, *N*-acetyl glucosamine; red triangle, fucose; purple diamonds, *N*-acetylneuraminic acid.

**
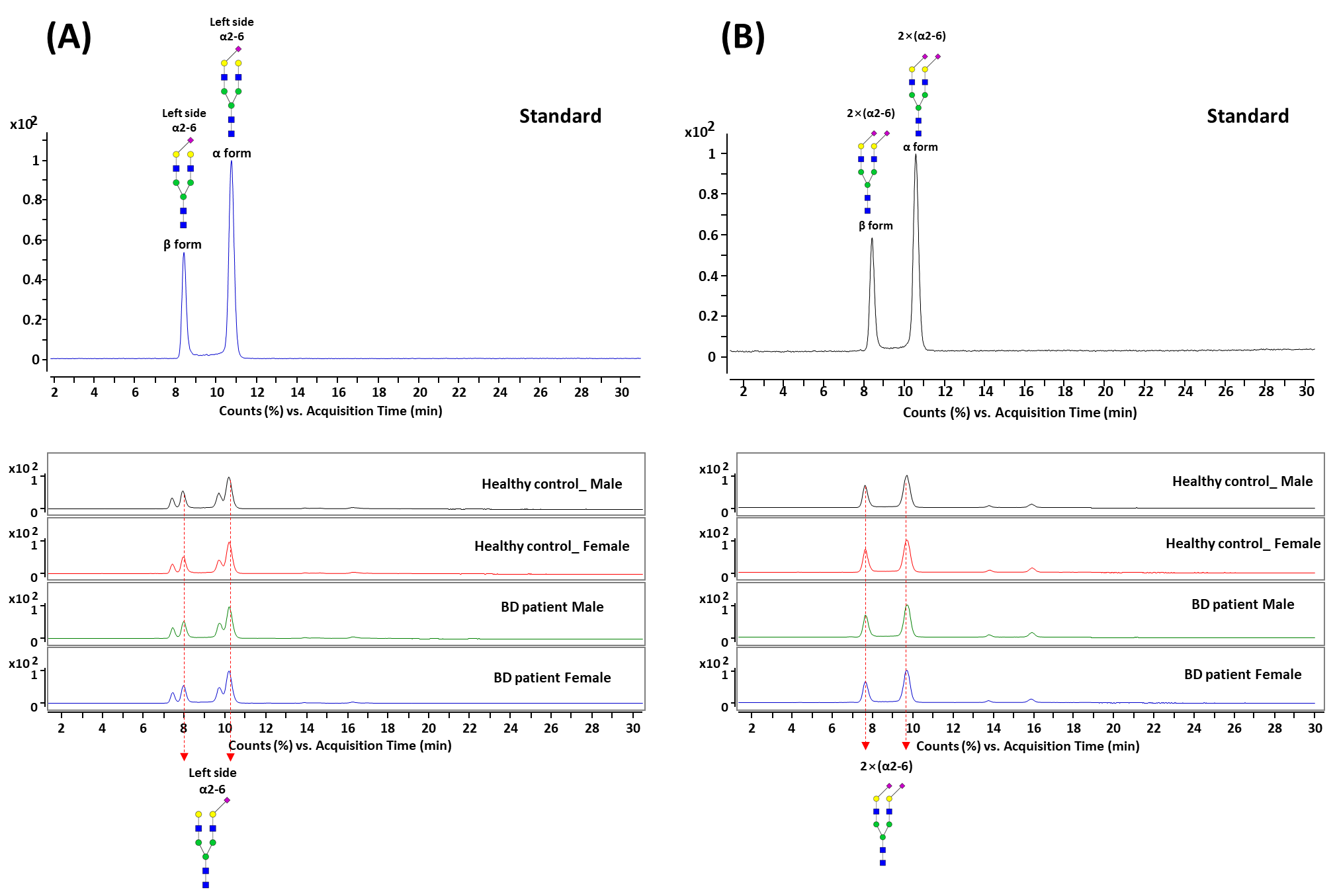
**

**Figure S4.** Scatter plots showing the distribution of absolute abundance of sialylated glycan isomers containing α2,3-sialic acid corresponding to (A) Hex_5_HexNAc_4_NeuAc_1_-1, (B) Hex_5_HexNAc_4_NeuAc_1_-2, and (C) Hex_5_HexNAc_4_NeuAc_2_-1. Top panels are comparison of male and female in healthy control group (Blue: male and Green: female), and bottom panels are comparison of healthy controls and Behcet’s disease patients in female (Green: control and Red: Behcet’s disease patient).


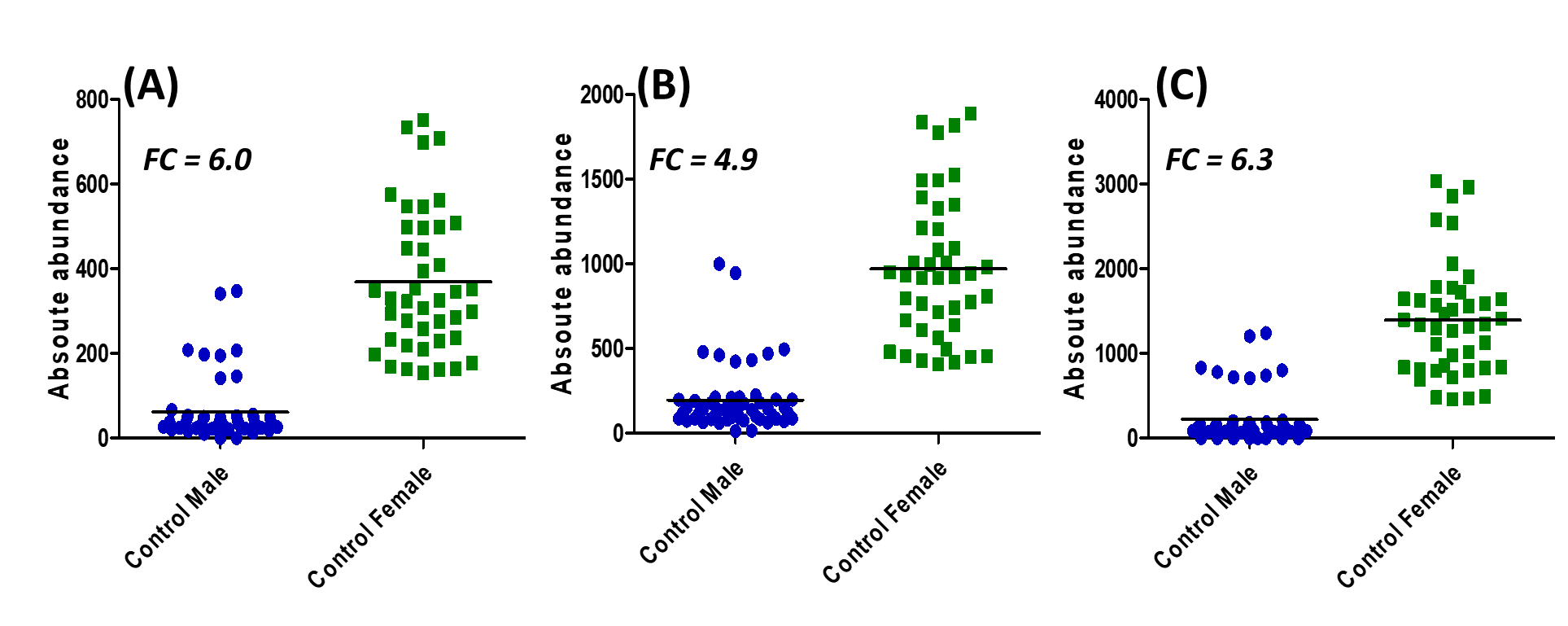

$$* Fold change \left( \mathrm{FC} \right)=\frac{average abundance of control female}{average abundance of control male}$$

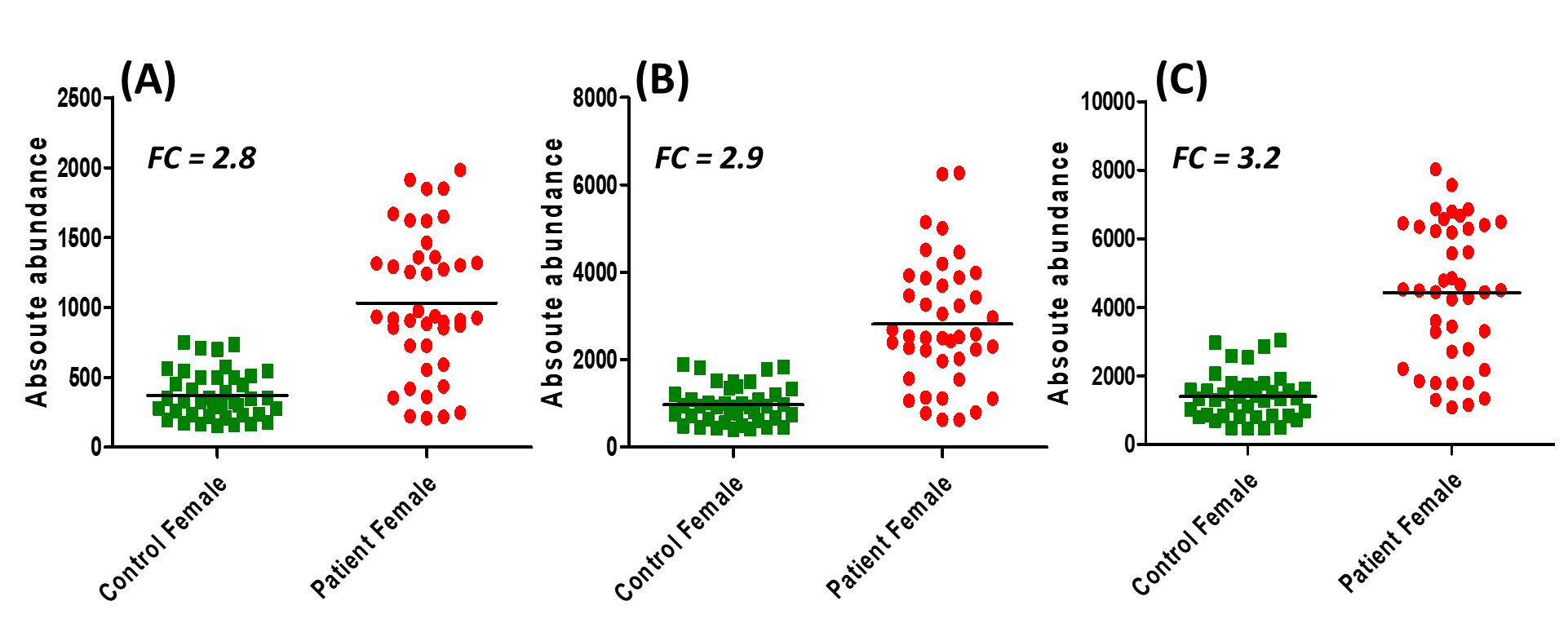

$$* Fold change \left( \mathrm{FC} \right)=\frac{average abundance of patient female}{average abundance of control female}$$

**Figure S5.** (A) total ion chromatogram (TIC), (B) mass spectra, and (C) CID-tandem mass spectra for three sialylated glycan isomers corresponding to Hex_5_HexNAc_4_NeuAc_1_, Hex_5_HexNAc_4_Fuc_1_NeuAc_1_, and Hex_5_HexNAc_4_NeuAc_2_. Glycan schematic symbols: green circle, mannose; yellow circle, galactose; blue square, *N*-acetyl glucosamine; red triangle, fucose; purple diamonds, *N*-acetylneuraminic acid.


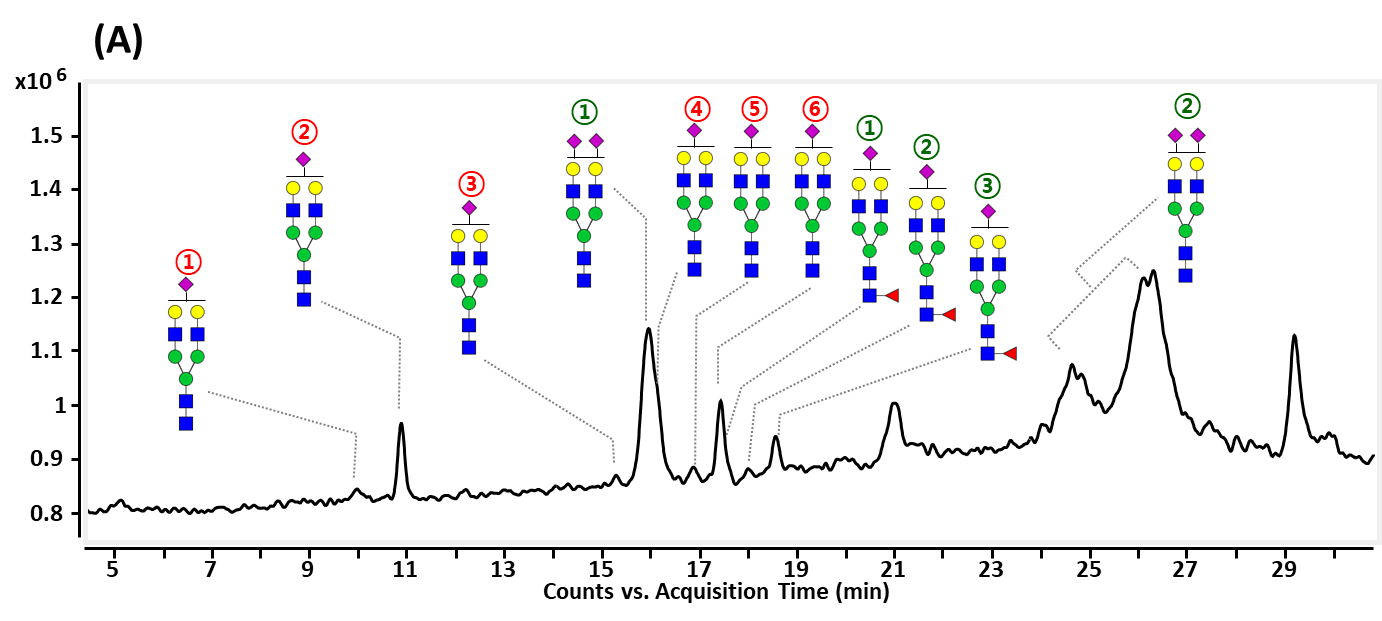


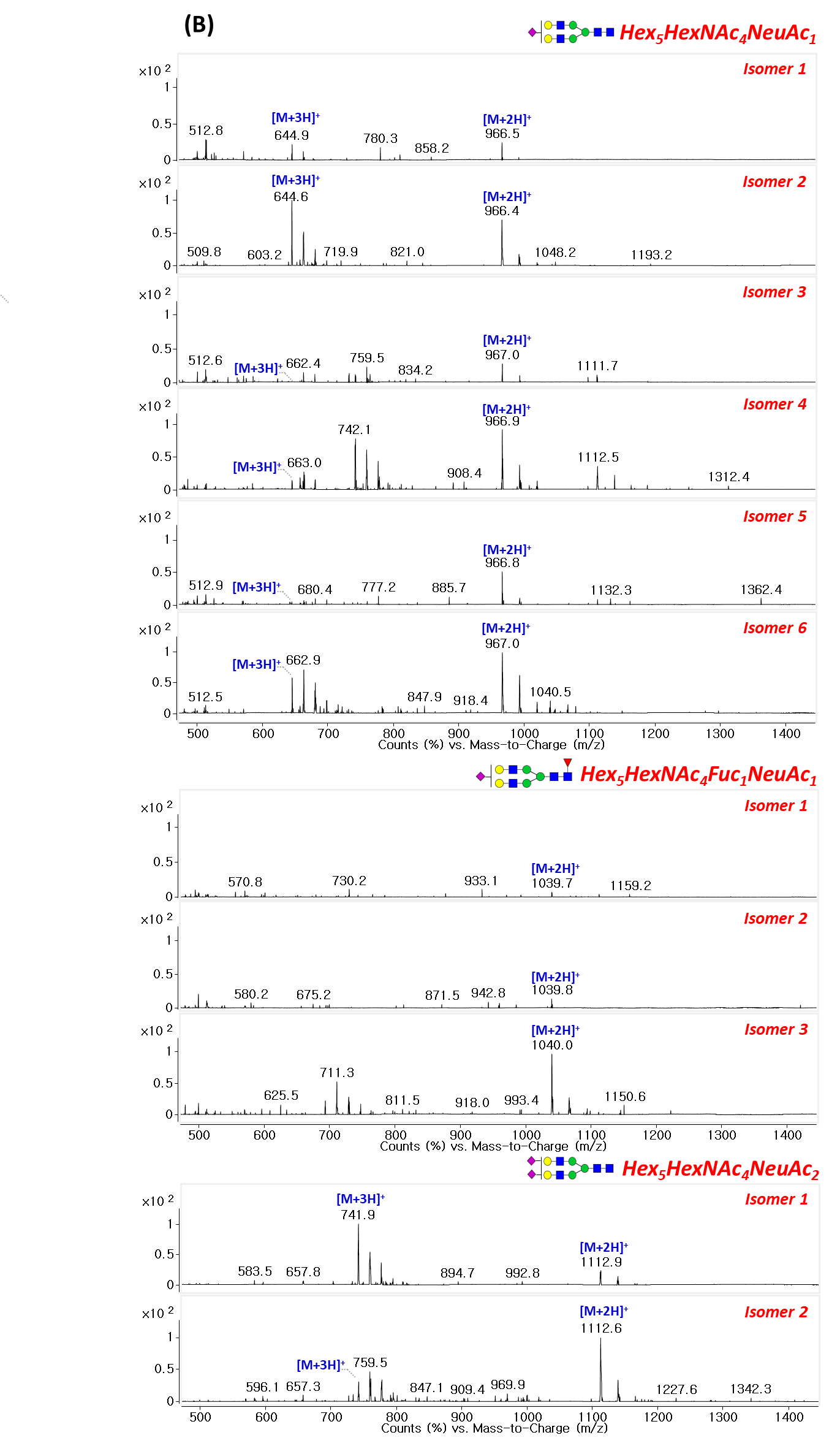


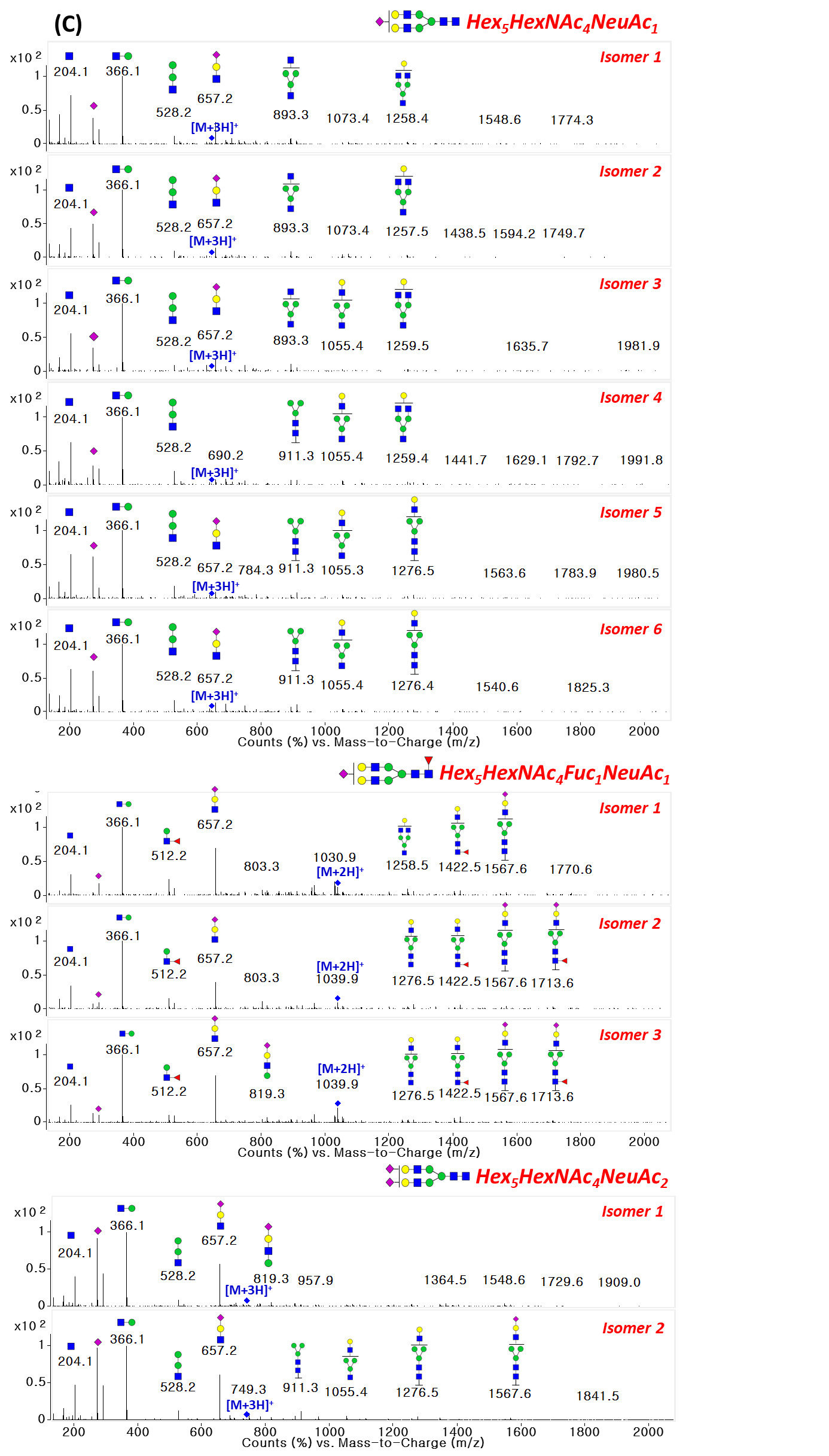

Supplement: Supplementary file 1 [file DataSheet1.docx]
